# Role of hydrogen bond alternation and charge transfer states in photoactivation of the Orange Carotenoid Protein

**DOI:** 10.1038/s42003-021-02022-3

**Published:** 2021-05-10

**Authors:** Igor A. Yaroshevich, Eugene G. Maksimov, Nikolai N. Sluchanko, Dmitry V. Zlenko, Alexey V. Stepanov, Ekaterina A. Slutskaya, Yury B. Slonimskiy, Viacheslav S. Botnarevskii, Alina Remeeva, Ivan Gushchin, Kirill Kovalev, Valentin I. Gordeliy, Ivan V. Shelaev, Fedor E. Gostev, Dmitry Khakhulin, Vladimir V. Poddubnyy, Timofey S. Gostev, Dmitry A. Cherepanov, Tomáš Polívka, Miroslav Kloz, Thomas Friedrich, Vladimir Z. Paschenko, Victor A. Nadtochenko, Andrew B. Rubin, Mikhail P. Kirpichnikov

**Affiliations:** 1 Faculty of Biology, M.V. Lomonosov Moscow State University, Moscow, Russia; 2 A.N. Bach Institute of Biochemistry, Federal Research Center of Biotechnology of the Russian Academy of Sciences, Moscow, Russia; 3 M.M. Shemyakin and Yu.A. Ovchinnikov Institute of Bioorganic Chemistry, Russian Academy of Sciences, Moscow, Russia; 4 Research Center for Molecular Mechanisms of Aging and Age-Related Diseases, Moscow Institute of Physics and Technology, Dolgoprudny, Russia; 5 Institut de Biologie Structurale J.-P. Ebel, Université Grenoble Alpes-CEA-CNRS, Grenoble, France; 6 Institute of Biological Information Processing (IBI-7: Structural Biochemistry), Forschungszentrum Jülich, Jülich, Germany; 7 JuStruct: Jülich Center for Structural Biology, Forschungszentrum Jülich, Jülich, Germany; 8 Institute of Crystallography, RWTH Aachen University, Aachen, Germany; 9 N.N. Semenov Federal Research Center for Chemical Physics, Russian Academy of Sciences, Moscow, Russia; 10 European XFEL GmbH, Schenefeld, Germany; 11 Chemistry department, M.V. Lomonosov Moscow State University, Moscow, Russia; 12 A.N. Belozersky Institute of Physical-Chemical Biology, Moscow State University, Moscow, Russia; 13 Institute of Physics, Faculty of Science, University of South Bohemia, České Budějovice, Czech Republic; 14 ELI-Beamlines, Institute of Physics, Praha, Czech Republic; 15 Technische Universität Berlin, Institute of Chemistry PC14, Berlin, Germany

**Keywords:** Molecular biophysics, Proteins, X-ray crystallography

## Abstract

Here, we propose a possible photoactivation mechanism of a 35-kDa blue light-triggered photoreceptor, the Orange Carotenoid Protein (OCP), suggesting that the reaction involves the transient formation of a protonated ketocarotenoid (oxocarbenium cation) state. Taking advantage of engineering an OCP variant carrying the Y201W mutation, which shows superior spectroscopic and structural properties, it is shown that the presence of Trp201 augments the impact of one critical H-bond between the ketocarotenoid and the protein. This confers an unprecedented homogeneity of the dark-adapted OCP state and substantially increases the yield of the excited photoproduct S*, which is important for the productive photocycle to proceed. A 1.37 Å crystal structure of OCP Y201W combined with femtosecond time-resolved absorption spectroscopy, kinetic analysis, and deconvolution of the spectral intermediates, as well as extensive quantum chemical calculations incorporating the effect of the local electric field, highlighted the role of charge-transfer states during OCP photoconversion.

## Abstract (web summary)

Yaroshevich et al. present a chemical reaction mechanism of a 35-kDa blue light-triggered photoreceptor, the Orange Carotenoid Protein (OCP). They find that photoactivation critically involves the transient formation of a protonated ketocarotenoid (oxocarbenium cation) state. This study suggests the role of charge-transfer states during OCP photoconversion.

## Introduction

In cyanobacteria, carotenoid-dependent non-photochemical quenching is mediated by a unique class of water-soluble carotenoid-binding proteins, the homologs of the Orange Carotenoid Protein (OCP). OCP was identified in 1981^1^, however, its structure^2^ and function^3–6^ were established only decades later. OCP is a 35-kDa photoreceptor^7^ triggered by blue light and coordinates a single ketocarotenoid molecule required for photoactivity. When the protein is purified from native OCP-containing cyanobacteria it is 3’-hydroxyechinenone^8^, but OCP is also fully functional with either echinenone (ECN) or canthaxanthin (CAN) instead. In the compact dark-adapted orange state (OCP^O^), the ketocarotenoid is enclosed by two protein domains of about equal size: an all α-helical N-terminal domain (NTD) and a mixed α-helical/β-sheet C-terminal domain (CTD)^2^. Upon photoactivation, OCP converts into the physiologically active red state (OCP^R^) via the formation of numerous intermediates^9,10^, and the quantum yield of OCP^R^ formation is extremely low (about 0.2%)^7,11,12^. During the conversion into OCP^R^, the structure of OCP undergoes global rearrangement: the domains separate from each other, after the carotenoid moves 12 Å deeper into the NTD^13–15^. These events lead to the exposure of sites for protein-protein interactions with the light-harvesting antenna complex, the phycobilisome (PBS), enabling quenching of its excitation^16–19^. In such a way, OCP^R^ prevents the overexcitation of the photosynthetic reaction centers and efficiently suppresses the photodamage of the photosynthetic apparatus. Under in vitro conditions in the absence of PBS, OCP^R^ spontaneously back-converts into OCP^O^ in the dark, which is strictly dependent on temperature^11,12,20^. Termination of the OCP-dependent PBS fluorescence quenching in vivo is usually promoted by the Fluorescence Recovery Protein (FRP), which forces detachment of OCP^R^ from PBS and promotes relaxation of OCP^R^ back to OCP^O^^18,21–23^. Despite recent advances in characterization of protein-protein interactions of OCP with FRP and PBS^24,25^, two crucial questions remain unanswered: (i) how does OCP manage to dissipate excitation energy of PBS pigments, and (ii) how does photoexcitation of the carotenoid in OCP trigger its activation? The key to both questions may emerge from the understanding of enigmatic excited states of the carotenoid in OCP.

Carotenoids exhibit exceedingly complex excited-state dynamics allowing them to play a key role in numerous processes such as light-harvesting, photoprotection, antioxidative defense including scavenging of reactive oxygen species (ROS)^26–28^. Carotenoids belong to the C_2h_ symmetry group, which is characterized by a two-fold rotational symmetry axis and one mirror plane perpendicular to this axis. The molecular symmetry considerations invoke strict selection rules, implying that the S_0_ (^1^A_g_) → S_1_ (^2^A_g_) transition is forbidden because both states have the same parity due to the presence of an inversion center. Such transitions were not observed even in the case of carotenoids embedded in proteins, for which the configurational asymmetry was directly indicated by strong circular dichroism in the visible range^29,30^. Thus, the first allowed transition of the lowest excitation energy is S_0_ (^1^A_g_) → S_2_ (^1^B_u_), which determines the absorption spectrum and usually covers the blue-green region of the visible spectrum (400–500 nm).

Femtosecond optical transient absorption spectroscopy is a powerful technique to examine the excited state dynamics of chromophores. This technique has been employed in numerous studies on carotenoids in various solvents and embedded in proteins^31^, including a series of studies on OCP-related proteins^9,32–37^. Transient absorption spectra of carotenoids usually exhibit negative amplitudes in the visible range, which are due to ground-state bleaching (GSB) in the course of the allowed S_0_-S_2_ excitation, and the characteristic S_1_–S_n_ excited state absorption (ESA), which occur after the very fast S_2_-S_1_ internal conversion (less than 100 femtoseconds) and vibrational cooling in the S_1_ state^38,39^. For linear carotenoids, the lifetime of S_1_ is inversely proportional to the number of π-electrons in the conjugated system of C=C bonds, which is essentially the consequence of the energy gap law^40^. This correlation is also observed in cyclic carotenoids such as β-carotene. However, since the end rings can be twisted relative to the plane of the conjugated chain, non-integer values are observed for the effective conjugation length (N), which indicates that double bonds in the end rings partially contribute to the conjugated system^41^. This notion can be further extended to ketocarotenoids such as echinenone, canthaxanthin, and 3′-hydroxyechinenone, since the carbonyl group might also partially contribute to the π-conjugated system^41^. In polar solvents, and especially when embedded in protein matrices, ketocarotenoids show additional features in femtosecond transient absorption spectra which are attributed to intramolecular charge transfer (ICT) states occurring farther red-shifted from the S_1_-S_n_ spectral signatures. ICT signatures are valuable indicators for the contribution of the carbonyl group(s) to the conjugated system, and might inform on the configuration of the carotenoid end rings^42,43^. These effects are of particular interest for the chromophores embedded in OCP since the only specific carotenoid-protein interactions in the OCP^O^ state are two short and strong hydrogen bonds between the 4-keto oxygen of the carotenoid and two hydrogens in the CTD, one belonging to the Tyr-201 hydroxyl group and another to the N-H group of Trp-288 (numbering corresponds to the OCP sequence of Synechocystis sp. PCC 6803). These hydrogen bonds affect the positioning of the terminal ring and decouple it from the conjugated polyene chain^41^.

Amino acid substitutions of both Tyr-201 and Trp-288 lead to destabilization of the compact OCP^O^ state and result in permanently red-shifted OCP forms (e.g., OCP^Y201A/W288A 12,21,44^, further designated using the one letter code OCP^AA^, as we focus on substitutions of Tyr-201 (first superscript letter) and Trp-288 (second superscript letter) in this work). Such red forms with separated protein domains are capable of inducing PBS fluorescence quenching without prior light activation^21^. However, many other amino acid substitutions lead to the same result due to destabilization of carotenoid-binding abilities of OCP, which increases conformational and thus spectral heterogeneity of the sample^20,45,46^. Spectral heterogeneity can also be increased due to the embedment of different carotenoids into OCP^47^. For example, the OCP^W288A^ variant (according to our nomenclature described above, OCP^YA^) expressed in ECN/CAN-producing *E. coli* strains preferentially bind CAN, appears red-purple and is not photoactive, although WT OCP with CAN is orange (in the dark-adapted state) and photoactive^45^. At the same time, even in the dark-adapted OCP several experimental approaches revealed spectral heterogeneity and a contribution from red-like forms confined in the compact protein structure^36,41,48^. Consistent with this, modelling of the linear absorption spectrum of dark-adapted WT OCP requires consideration of a red-shifted OCP^R^-like component^41^. This suggests that the carotenoid can principally occur in several distinct configurations in the compact OCP state.

Sample heterogeneity together with the low quantum yield of OCP photoconversion makes it difficult to spot the formation of photoproducts in pump-probe experiments. Only recent developments of ultrafast spectroscopic approaches revealed the existence of so-called S* features^49^ in OCP with a yield of about 5%, which may represent either a structurally distorted form of the S_1_ carotenoid state^9^ or a hot ground state with extended lifetimes^39,49,50^. Femtosecond pump-probe fluorescence spectroscopy of an OCP variant with only one critical tryptophan residue (Trp-288) left in place revealed that the hydrogen bond with the carotenoid disappears with a time constant (~20 ps) corresponding to the S* state lifetime, suggesting that the S* state might be related to breakage of the hydrogen bond(s) and formation of the very first intermediate of the OCP photocycle^10^. Since the breakage of the hydrogen bonds between the keto oxygen and Tyr-201/Trp-288 residues is considered to be a crucial step towards the activation of OCP, a detailed analysis of this reaction is necessary for understanding the mechanism of OCP photoconversion.

In this work, we present our approach to elucidate the impact of the aforementioned hydrogen bond donors on the spectroscopic properties and photoactivity of OCP and infer structural determinants of spectral heterogeneity. After selection and atomic structure determination of the most spectrally homogeneous OCP sequence variant available, a construct termed OCP^WW^, we carried out femtosecond transient absorption spectroscopy experiments. The OCP^WW^ construct exhibits striking differences compared to wild-type OCP regarding excited state dynamics with accentuated occurrence of S* signatures. Based on experimental data, computational modeling and quantum chemical studies, we propose the mechanism of hydrogen bonds breakage upon photoexcitation of OCP.

## Results and discussion

### Spectral heterogeneity of OCP preparations is associated with the H-bond donors in positions 201 and 288

It is known that interruption of protein-carotenoid interactions by amino acid substitutions destabilizes the compact OCP state, facilitating the formation of additional protein forms and thereby increasing spectral heterogeneity^12,22,46,51^. Since photoactivity is an exclusive feature of the compact orange OCP state, this state should be considered a starting point of the photocycle. The increased hydrodynamic size of the apoform and of red forms with separated protein domains allows for a complete chromatographic separation of these expanded forms from the compact holoform of OCP^21,22^. In order to exclude other intermediates from consideration, we sought for spectrally homogenous OCP variants, which would ideally exhibit only OCP^O^ features in the dark-adapted state, without any signatures of the red forms.

In previous work^12^, we demonstrated that the purple CAN-containing OCP^YA^ variant could be converted into the orange photoactive state by kosmotropic agents (e.g., 0.8 M phosphate), which promoted compaction of the species with separated domains by reinforcing domain interactions. Of note, this was accompanied by the emergence of discernible vibronic structure in the carotenoid absorption spectrum of OCP^YA^. We suggested that this effect might be due to the reduction of the number of hydrogen bond donors in the CTD. This assumption was supported by the facts that (i) kosmotropes do not affect absorption of WT OCP and that (ii) a minor orange photoactive fraction of OCP^YA^ binding ECN exhibits intense vibronic bands even at low (0.2 M) concentrations of phosphate^12^. Therefore, it is safe to conclude that the cause of spectral heterogeneity resides within the CTD of OCP, and, more specifically, concerns the carotenoid-contacting residues in positions 201 and 288.

**Fig. 1:**
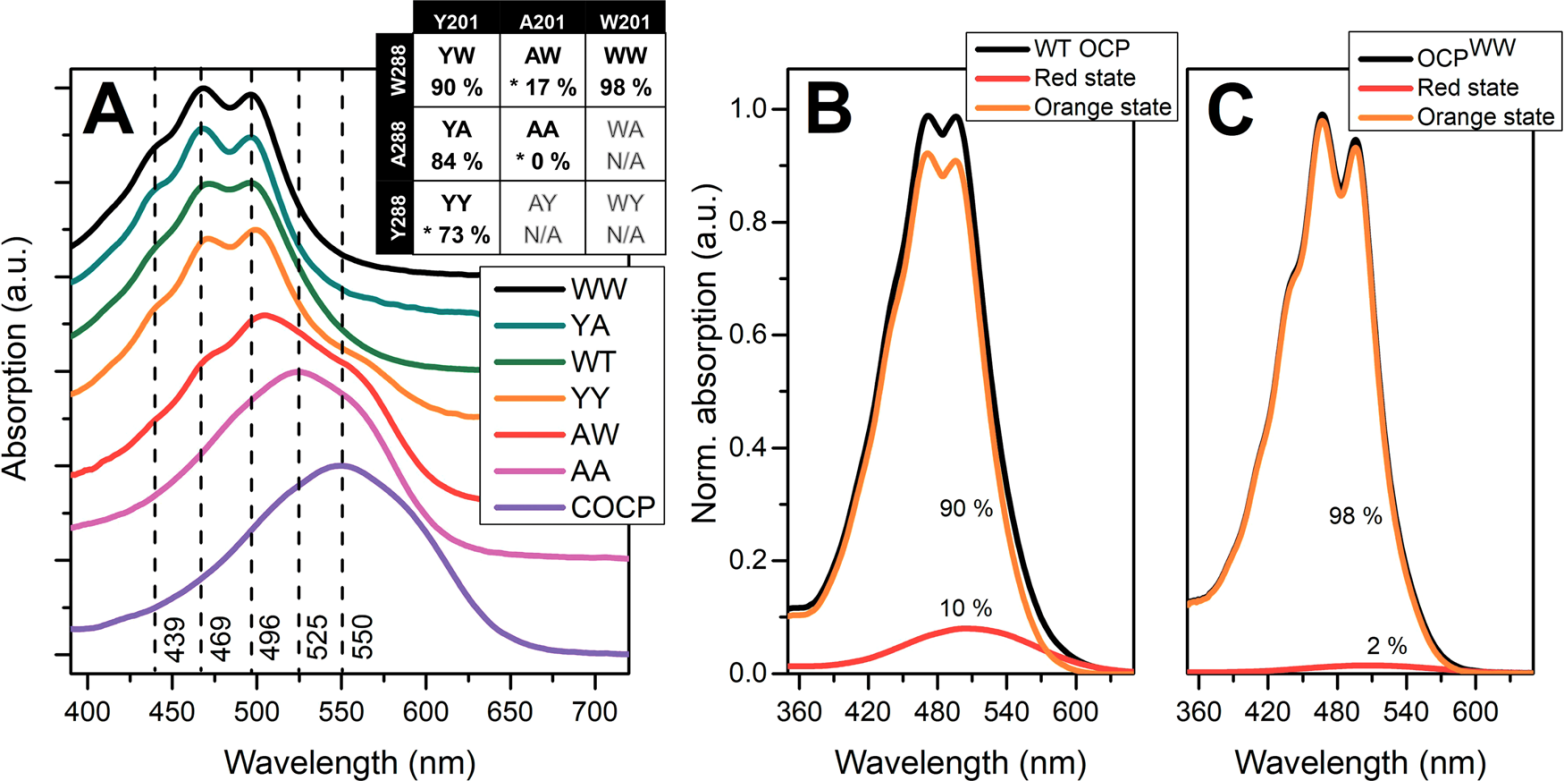
Variability of carotenoid absorption in OCP and its variants. **A** Absorption spectra of OCP and sequence variants thereof in 0.8 M phosphate. The inset shows the fractional content of the orange photoactive form. Asterisks represent OCP species not forming the orange state upon expression and purification from ECN/CAN producing *E. coli* strains; the orange compact forms of CAN-containing OCP^YY^ and OCP^AW^ appeared only in 0.8 M phosphate. “COCP” corresponds to the individual OCP-CTD forming a dimer coordinating CAN. **B**, **C** decomposition of the absorption spectra of the compact forms of WT OCP (**B**) and the OCP^WW^ variant (**C**) corresponding to the central positions of the elution peaks (apparent Mw ~35 kDa) on analytical SEC with full-spectrum detection. To estimate the yield of distinct spectral states, the absorption spectrum of the photoactivated protein was subtracted from the dark-adapted sample with an appropriate scaling factor. Numbers indicate the percentage of the orange and red states.

Spectral heterogeneity of the compact orange OCP state might be related to the competition of Trp-288 and Tyr-201 for H-bond formation to the keto oxygen of the carotenoid because of steric effects. Therefore, our strategy was to survey substitutions of these residues for their ability to affect the stability and spectral properties of OCP in the compact state (see Fig. 1). Importantly, a compact state could not be formed in the absence of both H-bond donors (in the OCP^AA^ sequence variant carrying Y201A/W288A substitutions). In all other cases, an orange photoactive species could in principle be obtained, albeit with a considerably different yield (Fig. 1A). The OCP^YY^ (W288Y substitution) and OCP^AW^ (Y201A substitution) variants expressed in ECN/CAN-producing *E. coli* strains bound exclusively CAN and showed no features of orange photoactive states in the absence of kosmotropes, which indicates that the stability of the compact state in these constructs is low. The OCP^WW^ (Y201W substitution) and OCP^YA^ (W288A substitution) variants were able to bind ECN as well, and, therefore, the orange compact state (with ECN bound) could efficiently be separated from the apoprotein and from purple, CAN-binding forms by size-exclusion chromatography (SEC). While the stability of the compact state of OCP^YA^ was still low compared to WT, the unprecedently stable OCP^WW^ variant was seen as a very fortunate object for the purpose of our investigation.

OCP^WW^ is capable of forming almost exclusively the compact orange state and is the most stable among all variants investigated. Its compact state is spectrally and structurally almost free from expanded OCP^R^-like states (Fig. 1C), which makes it the best candidate from our portfolio (Fig. 1A) for spectroscopic and structural studies. However, careful inspection of the absorption spectra of different OCP variants also reveals heterogeneity of their extreme orange states. In particular, the vibronic structure is least pronounced in WT OCP and most pronounced in OCP^YA^ (in which the compact state is not stable, note the ~16% contamination by OCP^R^-like states), while the absorption spectrum of OCP^WW^ is intermediate between these two (Fig. 1A and Supplementary Fig. 1). Therefore, we questioned if the tryptophan residue introduced in position 201 actually forms a hydrogen bond with the ketocarotenoid in the OCP^WW^ variant.

### Atomic structure of OCP^WW^ reveals peculiarities of protein-pigment interactions

**Fig. 2:**
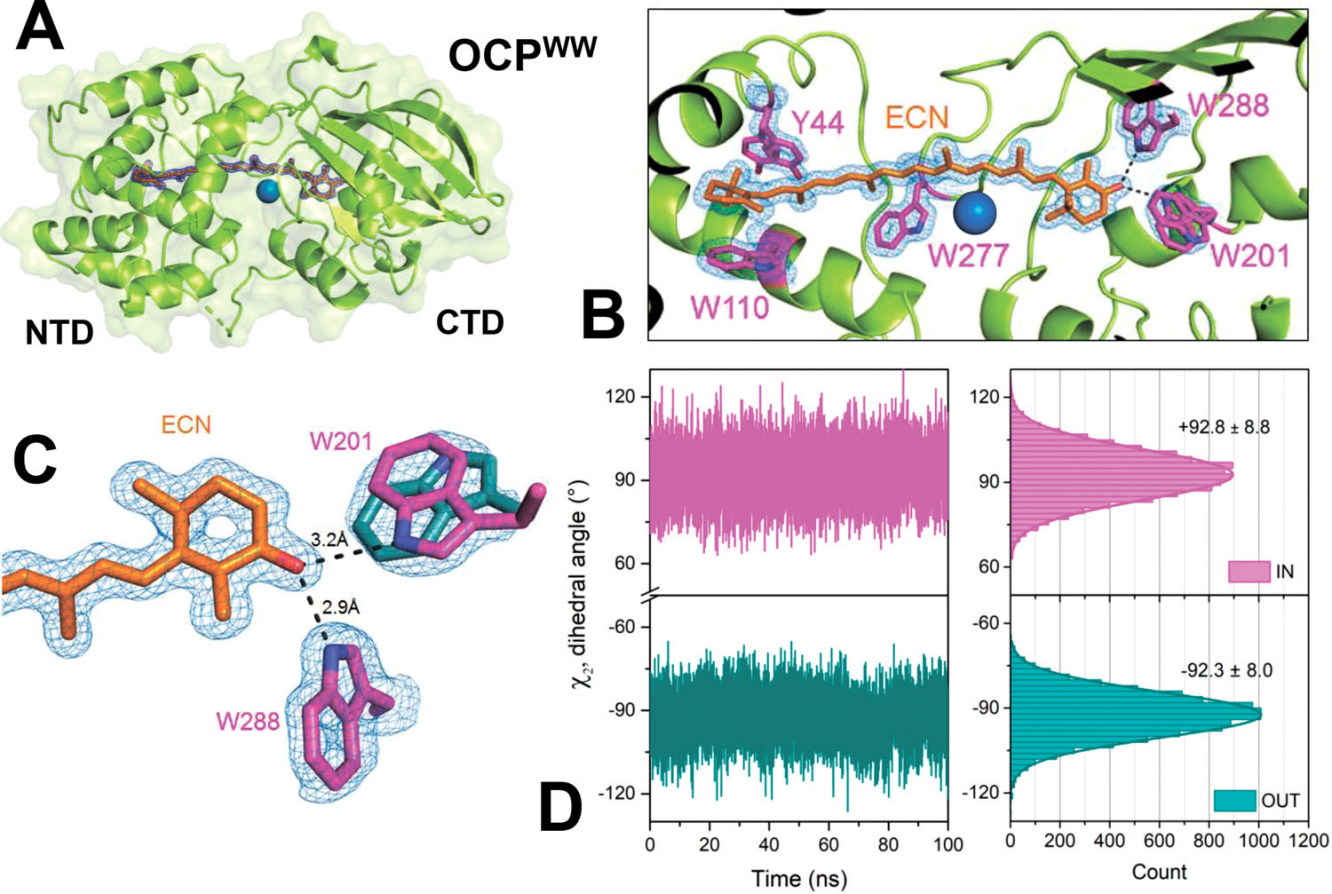
The most complete, representative crystal structure of the Y201W variant of OCP (OCP^WW^), PDB ID 6T6O. **A** Overall structure of ECN-binding OCP^WW^ shown as a cartoon backbone and a semi-transparent surface. A chloride ion is shown as a blue sphere. **B** Close-up view on the carotenoid-binding pocket of OCP^WW^. Crucial amino acids with hydrophobic side chains are shown in purple. Note the alternative positions of Trp-201 and Tyr-44. The other structures determined at different pH values (PDB IDs 6T6K, 6T6M) also reveal two alternative conformations for these amino acids. **C** Organization of hydrogen bonds between the keto oxygen of ECN and Trp-288/Trp-201. Panels **A**–**C** show 2F_O_-F_C_ electron density maps contoured at 3σ (**A**) or 1σ (**B**, **C**). **D** Values of the dihedral angle (χ_2_) between the peptide backbone and the indole ring of Trp-201 as revealed by MD simulations of OCP^WW^ with different initial conformation of Trp-201 (as shown in panel **C**: IN – purple, OUT – cyan).

To elucidate possible reasons behind the spectral heterogeneity of OCP in its compact orange state, we determined the atomic structure of OCP^WW^. It crystallized at three different pH values in the same space group P3_2_21, giving three structures with resolutions from 1.37 Å (pH 6.5) to 1.49 Å (pH 5.5) (Table 1 and Supplementary Table 1). We would like to note that OCP is stable and photoactive even at the lowest pH used for crystallization (4.6, see Supplementary Fig. 7 and description). All three structures confirm binding of exclusively ECN, are well superimposable, and reveal a protein fold that is barely distinguishable from that of WT OCP (Cα RMSD is 0.15–0.19 Å upon overlay with the PDB ID 4XB5 structure^15^, Supplementary Fig. 2). The position and conformation of Trp-288 are identical in WT and OCP^WW^, indicating the presence of a strong hydrogen bond between the keto oxygen and Trp-288 (the distance between the carotenoid’s keto oxygen and the nitrogen of Trp-288 is 2.9 Å). The electron density for the residue 201 reveals two alternative conformations of the engineered Trp in this position with nearly identical occupancies, nicely supported at this high level of spatial resolution. One Trp-201 conformation has the nitrogen oriented towards the keto group of the carotenoid (‘IN’ conformation), presumably enabling the formation of a weaker hydrogen bond with a distance of 3.2 Å. In the second Trp-201 conformation (‘OUT’), the side chain is rotated by 180° roughly in the same plane, which is incompatible with a hydrogen bond to the keto oxygen of ECN (Fig. 2C). Analysis of F_o_-F_o_ difference maps shows that IN and OUT Trp-201 rotamers have identical occupancy (within experimental error) each at all probed pHs. The distribution of dihedral angles (χ_2_) along the obtained molecular dynamics (MD) simulation trajectories shows similar mobility of Trp-201 conformation ‘IN’ and ‘OUT’ states on the ns time-scale (Fig. 2D). MD simulations of the OCP^WW^ structures starting from these two distinct orientations of the Trp-201 side chain (“IN” or “OUT”) revealed no transitions between these two conformations in two independent 100 ns-long trajectories (Fig. 2D). This hints at a low rate of transitions between these forms at least in the basal dark-adapted OCP^WW^.

**Table 1:** OCP^WW^ X-ray data collection and refinement statistics.

	pH 6.5	pH 5.5	pH 4.6
PDB ID	6T6K	6T6M	6T6O
Data collection			
Space group	P 3_2_ 2 1	P 3_2_ 2 1	P 3_2_ 2 1
Cell dimensions: a, b, c (Å)	82.726, 82.726, 88.044	83.149, 83.149, 87.488	82.996, 82.996, 88.123
α, β, γ (°)	90, 90, 120	90, 90, 120	90, 90, 120
Resolution range (Å)*	44.02–1.37 [44.02–7.38] (1.39–1.37)	41.57–1.49 [41.57–8.16] (1.52–1.49)	41.50–1.40 [42–7.67] (1.42–1.40)
Wavelength (Å)	0.976250	0.976250	0.976250
R_merge_**	0.046 [0.038] (1.49)	0.038 [0.032] (1.21)	0.033 [0.031] (1.16)
R_meas_	0.048 [0.041] (1.57)	0.04 [0.034] (1.268)	0.034 [0.033] (1.193)
<I/σ>	29.1 (2.3)	37.3 (3.3)	41.4 (3.5)
CC_1/2_	0.999 (0.816)	0.999 (0.880)	1.00 (0.882)
Completeness (%)	100 (100)	100 (100)	100 (100)
Redundancy	20.2 (20.2)	20.2 (20.2)	20.0 (20.2)
Refinement			
Resolution range, (Å)	37.54–1.37	41.57–1.49	41.53–1.40
No. of reflections: total	69781	54626	65885
‘free’ set	3654	2826	3461
R_work_, (%)	13.5	12.84	12.84
R_free_, (%)	16.7	16.72	16.65
Average B-factor (overall Å^2^)	32.7	35.8	33.1
No. of non-H atoms: protein/ligands/solvent	2431/86/322	2438/51/343	2448/58/401
R.m.s.d. bond lengths (Å)/angles (°)	0.010/1.30	0.013/1.48	0.013/1.46
Ramachandran favored/outliers (%)	100/0.0	99.7/0.0	99.4/0.0
Molprobity score/Clash score	1.45/7.5	1.43/7.9	1.42/7.7

^*^Statistics for the lowest and highest resolution shells are indicated in square brackets and parentheses, respectively. The IN and OUT Trp-201 rotamers have 50% occupancy (within experimental error) each at all probed solution pH values.

Considering the similarity of the absorption spectra of OCP variants having only one hydrogen bond donor (i.e., the OCP^YA^ and OCP^AW^ mutants, see Fig. 1A and Supplementary Fig. 1) to that of OCP^WW^, we associate the manifestation of the vibronic features of ECN S_0_-S_2_ absorption in OCP^WW^ with the ‘OUT’ Trp-201 conformation, in which only one hydrogen bond is left (between ECN = O and Trp-288). If two hydrogen bonds are present simultaneously, in OCP^WW^ with the ‘IN’ Trp-201 conformation, we assume that absorption of this form should be similar to that of WT OCP (i.e., with the less pronounced vibronic structure), with the caveat that the length of the ECN = O···Trp-201 bond (3.2 Å) is appreciably longer than in the case of ECN = O···Tyr-201 in WT OCP (2.6 Å, see Supplementary Fig. 2). Although we noticed that a minor fraction of OCP^WW^ indeed has absorption similar to WT OCP, a major part of the sample has very profound vibronic features (Fig. 1A and Supplementary Fig. 1). Thus, due to the long H-bond (3.2 Å), which in addition is formed only in half of the observed situations, the interaction of the carotenoid with Trp-201 in OCP^WW^ appears to be weak and likely has limited effects on ECN absorption. This suggests that the single H-bond between ECN = O and Trp-288 is dominating and is responsible for the profound vibronic structure in the absorbance spectrum. Following this logic, we assume that the orange state with only one hydrogen bond is present also in OCP^YY^, because Tyr in place of Trp-288 would not be suitable for the formation of a strong hydrogen bond with ECN = O. We assume that the absence of the second, weak donor of the hydrogen bond in position 201 accentuates the role of Trp-288 in protein-pigment interactions in OCP^WW^.

Additionally, our structures also revealed heterogeneity of the Tyr-44 conformation (Fig. 2B). It is known that substitution of this residue by serine affects photoactivity of OCP, making photoinduced accumulation of the physiologically active red state ineffective^44^, most likely due to an increased rate of back conversion. Therefore, besides characterization of the uniquely homogeneous absorption spectrum of OCP^WW^, it was necessary to analyze OCP^WW^ in terms of photoactivity.

### Photoactivity of OCP^WW^ reveals a reduced number of hydrogen bonds with ketocarotenoid in solution

To test the ability of OCP^WW^ to form the red state upon photoactivation, we performed a series of photoconversion kinetics experiments with relatively long (10 s) exposure to actinic light (see^11^ for description) at different temperatures, for comparison with WT OCP and OCP^YA^. Upon illumination of the dark-adapted OCP^WW^ sample by actinic blue light (450 nm, LED 200 mW), we observed a gradual increase of the optical density at 550 nm, which was completely reversible in the dark, although back-conversion was considerably slower compared to WT OCP (Supplementary Fig. 3). Such a reduction of the back-conversion rate in OCP^WW^, which is also observed in the OCP^YA^ sample, can be explained by a reduced number of residues involved in the anchoring of the ECN keto group upon the translocation of carotenoid back into the CTD. Since the activation energies (see Table 2) for the back-conversion of photoactivated mutants (OCP^WW^ and OCP^YA^) are comparable to the one of WT OCP, we assume that the lack of one hydrogen bond donor results in an increased number of spontaneous conformational motions during achievement of the basal conformational state. These extended carotenoid and protein configurational dynamics decrease the probability of reformation of the compact orange state and, therefore, reduces the corresponding rate of the red state relaxation. Using a kinetic model proposed earlier^11^, we determined the apparent activation energy necessary for the transition from the orange into the red state and found that in OCP^WW^ this reaction requires only 2.3 kcal/mol which is approximately 5 kcal/mol less compared to WT OCP (Table 2 and Supplementary Fig. 3). Remarkably, the corresponding apparent activation energy for the accumulation of the red state was also reduced in the OCP^YA^ sample compared to WT OCP. Since one hydrogen bond is also absent in the OCP^YA^ sample, we assume—due to the similarities of the observed effects on activation energies and the clearly monoexponential decay of the red state (Supplementary Fig. 3)—that the second H-bond is also partially absent in OCP^WW^. This is in line with our structural data (Fig. 2) showing that Trp-201 in the “OUT” orientation is not suitable for the formation of the hydrogen bond, while in the “IN” state the strength of the hydrogen bond must be lower than in OCP WT due to its increased length (3.2 Å vs. 2.6 Å). Thus, we see that the role of Trp-288 as a hydrogen bond donor in OCP^WW^ increases compared to that in WT OCP, in which the carotenoid shares hydrogen bonds with both, Trp-288 (2.9 Å) and Tyr-201 (2.6 Å).

**Table 2:** Apparent activation energies (*E*_A_) for the rate of accumulation (k_O-R_) and subsequent relaxation (k_R-O_) of the OCP^R^ state in WT OCP and two sequence variants studied.

	*E*_A_ (k_O-R_), kcal/mol	*E*_A_ (k_R-O_), kcal/mol
WT OCP	7.5 ± 0.3	31.8 ± 1.1
OCP^WW^	2.3 ± 0.4	34.3 ± 1.0
OCP^YA^	2.6 ± 0.3	38.2 ± 0.6

Average power of actinic LED light was set to 200 mW in all experiments. Experiments were conducted in a range of temperatures from 25 to 40 °C. Values are given as means ± S.D. and resulted from three measurements.

Thus, in solution OCP^WW^ represents a unique OCP variant with only one strong hydrogen bond between the keto oxygen of ECN and Trp-288, which is still capable to form a stable and compact orange state in the dark and to reversibly convert into the red state upon photoactivation. Further, taking advantage of the spectral homogeneity of ECN in OCP^WW^, we will focus on the dynamics of carotenoid excited states which may cause photoactivation.

### Excited-state dynamics and photochemistry of the carotenoid in OCP

Recent works proposed the importance of the so-called S* state of ECN in the photoactivation mechanism of OCP^9,37^. In ultrafast transient absorption experiments, the S* state of the carotenoid was usually assigned to a group of signals arising on the blue flange (~550–600 nm) of the S_1_-S_N_ absorption with lifetimes in a range from 2 to 20 ps^49^. Usually, the S_1_-S_N_ absorption features decay faster (typical lifetime for OCP ~3.5 ps^32^) than those of the S* state (~10 ps). Notably, the presence of the S* state in OCP was first reported only in 2019^9^. We assume that this is due to the complexity of the experimental approach and the low yield of S* in WT OCP. Very recently, it was reported that S* is more pronounced in OCPs from the OCP2 clade than in members of the OCP1 clade^52^, although the yield of S* was still less than 5%^37^. Because members of the OCP2 clade have a more profound vibronic structure of the S_0_-S_2_ spectra and faster accumulation of the red active state compared to OCP1 variants, it was concluded that the S* state could be necessary for the photoactivation^37^. Since (i) the steady-state absorption spectrum of the OCP^WW^ mutant exhibits one of the most pronounced fine structure patterns among all known ketocarotenoid-containing OCP-related proteins (Fig. 1), and (ii) it indeed undergoes conversion to the active, red state with an almost 5 kcal/mol lower apparent activation energy than WT OCP (see Table 2), we hypothesized that transient absorption spectroscopy of OCP^WW^ might reveal peculiarities of the S* state due to an increased yield.

**Fig. 3:**
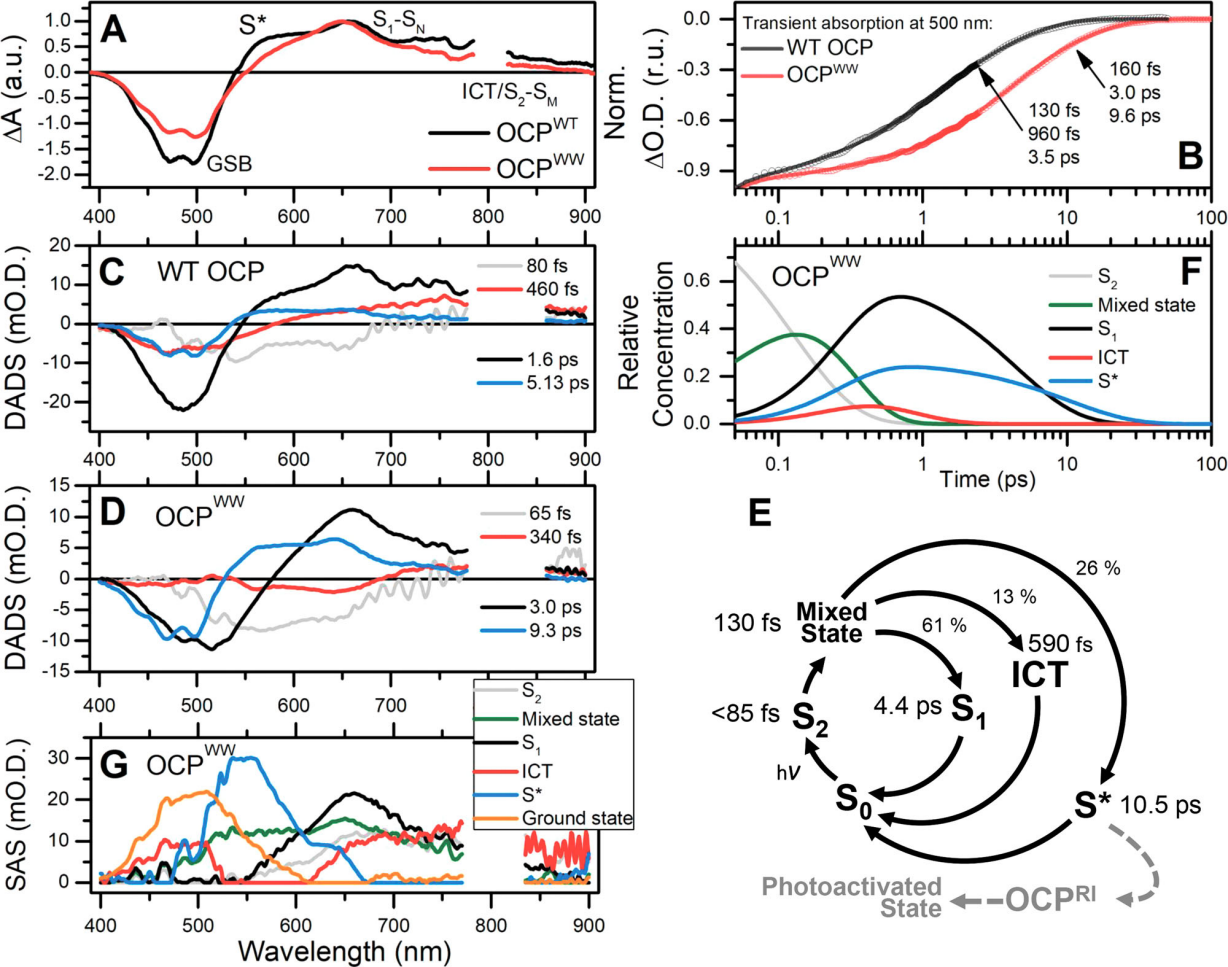
Excited state dynamics and photochemistry of the ketocarotenoid in OCP as revealed by transient absorption spectroscopy. **A** Transient absorption spectra of WT OCP (black) and OCP^WW^ (red). Spectra were recorded 1 ps after excitation with a 30-fs pulse at 520 nm for WT OCP and OCP^WW^. Spectra were normalized to the maximum of the ESA bands. **B** Time courses of transient absorption of WT OCP (black) and OCP^WW^ (red) measured at 500 nm probe wavelength. Numbers indicate the characteristic lifetimes of the corresponding states. Note the logarithmic timescale. **C**, **D** Decay-associated difference spectra (DADS) obtained from global fitting of the transient absorption spectra of WT OCP and OCP^WW^, respectively. **E** The model of transitions between the states of ECN in OCP after photoexcitation. Numbers indicate characteristic lifetimes of the corresponding states. OCP^RI^ represents a long-lived “red intermediate” OCP state, in which the protein structure is compact, while the conformation of the carotenoid is (quasi-)stabilized in a state with an increased conjugation length due to breakage of hydrogen bonds to the amino acid residues in the CTD. The following dashed arrow indicates possible slow transitions of OCP^RI^ into the physiologically active state OCP^R^. **F** Relative concentration kinetics of the states generated by a 26 fs pulse in OCP^WW^ and corresponding species-associated spectra (SAS) of the states (**G**) obtained from target analysis of transient absorption spectra of OCP^WW^ considering the model (**E**).

After excitation of carotenoid-containing proteins by 26 fs-long laser pump pulses at 520 nm, the transient absorption spectra of OCP reveal several distinct characteristic regions (Fig. 3A). Activation of the S_0_-S_2_ transition causes depopulation of the ground state which leads to the so-called ground state bleaching (GSB). Examination of the time courses of transient absorption in the GSB region reveals differences in excited state dynamics of WT OCP and OCP^WW^. Relaxation to the carotenoid ground state proceeds much slower in the mutant protein compared to WT OCP (see Fig. 3B). The excited state absorption (ESA) S_1_-S_N_ transition of ECN in WT OCP and OCP^WW^ is centered at approximately 650 nm. Since the S_0_-S_1_ transition is forbidden, S_1_ is formed by the decay of the S_2_ state with a lifetime of approximately 60–85 fs. Notably, the accumulation of ESA in the visible region occurs not only at the typical lifetime of S_2_, but also within 300–400 fs after the excitation, suggesting the presence of an intermediate state, which may decay into different long-living excited states (like S_1_). The S_2_–S_M_ transition dominates in ESA at wavelengths above 800 nm (up to 1200 nm, according to reference 37), which is only covered up to 910 nm by our present setup, thus we see only the blue flange as the positive band at 860–900 nm in the decay-associated difference spectra (DADS) component (Fig. 3D, gray curve), which is enough to estimate the lifetime of S_2_. Thus, during its short lifetime, S_2_ populates other excited states, and according to global analysis (Fig. 3C, D), ESA of OCP represents a superposition of at least four distinct components with different lifetimes. We assign the DADS component with approximately 3.0 ps lifetime, which has positive amplitude in the 600–800 nm region of the transient absorption spectra, to the mixture of the S_1_ state and a so-called Intramolecular Charge Transfer (ICT) state, which is characteristic of asymmetric ketocarotenoids in a polar environment, being especially pronounced in OCP at wavelengths above 700 nm^9,32–37,53–55^. The most general explanation of the ICT phenomenon is related to substantial transfer of the electron density in the excited states induced by the keto oxygen^56^. It should be noted that for many carotenoids, which do not show ICT signatures, ESA of S_1_ vanishes completely above 750 nm, which is not the case for the ~3.0 ps DADS component observed in our experiments (Fig. 3C, D, black curves). Thus, we assume that for WT OCP and OCP^WW^, ICT features are mixed with the S_1_ state.

In addition to presumable S_1_/ICT states in OCP^WW^, we observed a substantial contribution of a component decaying with a characteristic time constant of about 340 fs (Fig. 3D). This component has positive amplitude in DADS above 700 nm, and negative between 550 and 700 nm. Due to the pronounced positive ESA signal above 700 nm, we assign this component to an ICT-like state, which, as suggested by DADS, gives rise to the component with ~9.3 ps lifetime (Fig. 3D). Due to the aforementioned formalism, we assigned components with the longest lifetime to the S* state. The yield of the S* state is particularly high in OCP^WW^ (~25%), with spectral signatures dominating around 550 nm, while in WT OCP, the yield of components with lifetimes longer than 5 ps is relatively low. We would like to note an interesting feature in the S* spectrum—the high resolution of vibrational bands in the GSB region (Fig. 3C, D, blue lines). This strongly indicates that the S* signal is associated with a subset of OCP molecules with some special ground state configuration of ECN which is more pronounced in OCP^WW^ than in WT OCP. This might be related to a single hydrogen bond between ECN and protein in OCP^WW^ in ‘OUT’ configuration (see Fig. 2C).

Further, we conducted a target analysis of ESA in OCP^WW^, since it has the most pronounced S* features. The most suitable and explanatory kinetic model proposed (Fig. 3E) considers five states, including the so-called ‘Mixed State’ (or the hot S_1_/ICT/S* state), which is necessary to explain the relatively slow accumulation of S_1_, ICT, and S* signals after the rapid decay of the S_2_ state (see Fig. 3F). This model allowed us to reconstruct spectra of the individual states (Fig. 3G), separating features of S_1_, ICT, and S* that are superimposed in DADS. Absorption of the ‘Mixed State’ represents a broad group of bands, indicating that exited states S_1_, ICT and S* are formed already within the first 100 fs after excitation of the sample.

Thus, application of global and target analysis to transient absorption spectra of OCP^WW^, in which substitution of Tyr-201 by tryptophan increased the yield of the S* state at least fivefold compared to WT OCP, allowed us to disentangle the sequence of ultrafast photoinduced reactions, and to propose a comprehensive kinetic model, which places the S* state (Fig. 3E) in a critical position for proceeding towards the physiologically active red state. The model also indicates that the appearance of the S* state is associated with an intermediate state with ICT state features. In accordance, using ultrafast pump-probe fluorescence techniques applied to an engineered single-tryptophan OCP variant, we have recently found that breakage of the hydrogen bond between the keto oxygen of ECN and Trp-288 occurs with a time constant of 22.9 ± 2.0 ps^10^. This is in line with our suggestion that H-bond disruption must be initiated by transition of the carotenoid into a long-lived S* state. Further, we discuss the possible nature of these states and their role in the photoactivation of OCP.

### Possible mechanism of hydrogen bond dissociation upon OCP photoactivation

It is generally accepted that hydrogen bonding of ECN by Tyr-201 and Trp-288 prevents OCP from spontaneous transition into the active state^6,10,57,58^. However, it is well known that OCP activation can occur without photoexcitation of the carotenoid, and some OCP variants balance between the orange and active red state due to the reduced stability of their compact form^30,51^. Therefore, one cannot exclude processes of spontaneous hydrogen bond breaking under the influence of certain forces arising in the protein environment. But, since OCP is a photoreceptor, answering the question how the excitation energy absorbed by the carotenoid can force the breakage of hydrogen bonds is crucial. To address this question, we performed a series of quantum chemistry (QC) calculations to estimate the energetics of this process.

**Fig. 4:**
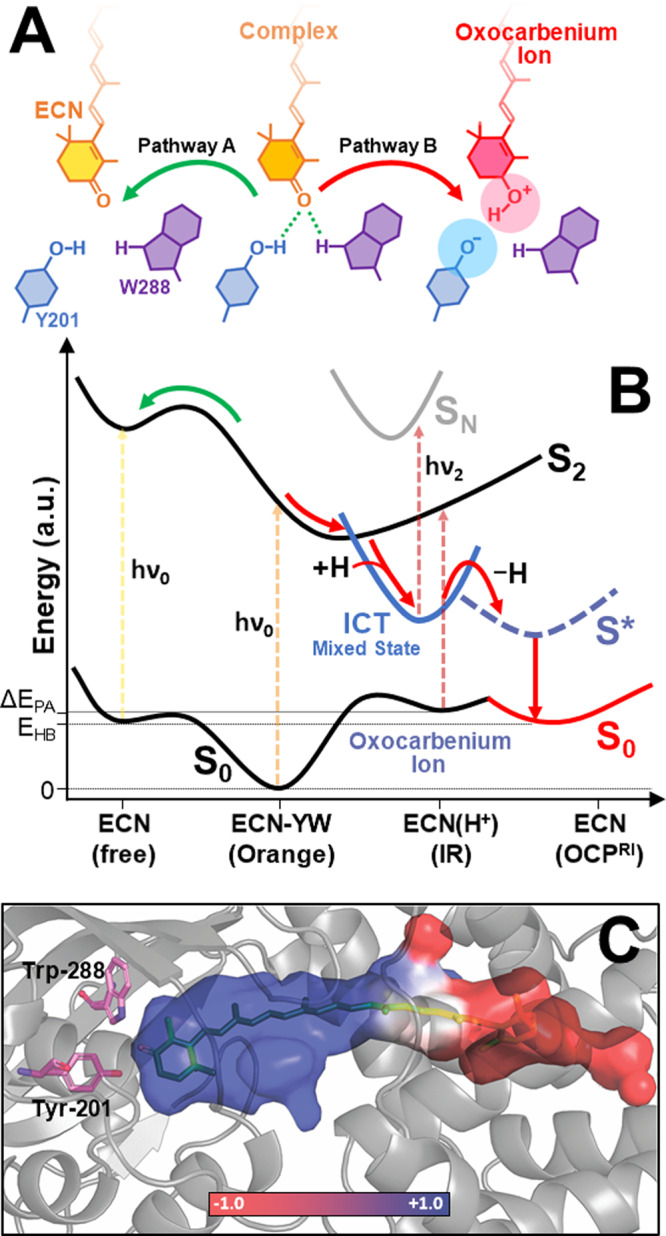
Proposed pathways of the hydrogen bond breakage in OCP. **A** Schematic configuration of ECN complexes with the conserved tyrosine (blue) and tryptophan (violet) residues. **B** Proposed scheme for the evolution of carotenoid excited electronic states in OCP. Green and red arrows show energy transitions in the system following coordinate A and B, respectively. The length of the dashed arrows shows the energy of light quanta absorbed in different states. **C** Distribution of the local electric field in the carotenoid-binding pocket of OCP in pseudo colors. To estimate the charge distribution in OCP, we used the PDB ID 3MG1 structure model with augmented hydrogen atoms and parameterized it with a partial charge formalism using CHARMM force field. Visualization of the electrostatic potential on the surface of the protein’s inner cavity was done with the apbs.tools plugin for PyMol. For more details see Supplementary Fig. 5 and its legend.

The energy of hydrogen bonds in the equilibrated ternary ECN/Tyr-201/Trp-288 complex (see Fig. 4A and Supplementary Fig. 4) in vacuum calculated with different DFT density functionals equals 13–15 kcal/mol (Supplementary Table 2–3). It is worth to mention that in OCP crystals, the geometry of this complex is far from the calculated energetically favorable conformation (especially regarding the position of Tyr-201, see Supplementary Table 4). According to our estimations, such a distortion decreases the hydrogen bond energy in OCP down to 8 kcal/mol (see Supplementary Table 3), which is in good agreement with our experimentally determined values for the apparent activation energy of the OCP^O^-OCP^R^ transition (see Table 2). Such an amount of energy is required to break hydrogen bonds and release ECN, Tyr-201, and Trp-288 as products (Fig. 4A, Reaction Pathway A). According to our calculations, the formation of hydrogen bond(s) between ECN and Tyr-201/Trp-288 causes a bathochromic shift of carotenoid absorption. Thus, dissociation of the hydrogen bond via Reaction Pathway A (Fig. 4A) would inevitably lead to a hypsochromic shift of the absorption spectrum of ECN (see Supplementary Table 5 for excitation energies for ECN and the ECN/Tyr-201/Trp-288 complex). However, this has never been reported in any femtosecond transient absorption experiment. The calculated energies of ECN in the excited states show that hydrogen bonds become about 3.5 kcal/mol stronger in the S_2_ state, while staying almost the same in S_1_ as in the ground state (Supplementary Table 5). Since hydrogen bond energy reduction in the excited states is not observed, it can be inferred that Reaction Pathway A is highly unlikely (see Fig. 4A, B) and alternative mechanisms should be considered.

Several hypotheses on the mechanism of photoinduced hydrogen bond breakage have been considered previously. Due to a different configuration of the carotenoid’s β-ring in crystals of full-length WT OCP and its NTD (the so-called red carotenoid protein, RCP) it was proposed^15^ that the C6 − C7 trans−cis isomerization happens during the initial stages of OCP photoactivation, however, no evidence for such a process was found on a ps timescale^9^. Thus, it is reasonable to assume that isomerization of the carotenoid occurs due to the difference in potential energy of trans (C6 − C7 dihedral angle ~ 130°) and cis conformation (~45°)^57^ in the absence of hydrogen bonds as a consequence of hydrogen bond breakage, but not as a prime cause for the breakage of the latter. Alternatively, keto-enol tautomerization of ECN in the excited state was proposed in^47^; however, such a mechanism is highly unlikely due to the huge energy difference between the hypothetical tautomers.

Thus, we consider an alternative mechanism for the disruption of the hydrogen bonds in the ECN/Tyr-201/Trp-288 complex involving charge separation (Fig. 4A, Coordinate B). Assuming a protonated ECN(H^+^) oxocarbenium cation and a deprotonated amino acid (a Tyr or Trp anion) as products, the energy of such a reaction could be estimated as the difference in proton affinity (PA) between the donor and the acceptor of the proton. Calculations show that the proton affinity of ECN in vacuo (see Supplementary Table 6) is lowest compared to the corresponding PA values of Trp or Tyr or a Tyr–Trp complex. The energy difference between the corresponding compounds, which is necessary to conduct the proton transfer, ranges from 82 to 106 kcal/mol (considering a Tyr–Trp complex or Trp as a hydrogen donor, respectively). This energy is extremely high even compared to the excitation by a blue photon (about 65 kcal/mol), suggesting that in vacuum such a reaction is impossible.

However, the proton affinity dramatically depends on the strength of the local electric field. For instance, a local environment with a negative charge will enhance Coulomb interaction with a proton, thereby causing an increase of the proton affinity for a compound. A peculiar feature of conjugated molecules is their high polarizability due to the vast delocalization of the π-electrons^59,60^. The distribution of the charged protein groups gives rise to an electrostatic potential gradient on the surface of the carotenoid-binding cavity of OCP ^61^. Noteworthily, the charge distribution within the carotenoid-binding cavity of OCP is highly asymmetric, with mostly positively charged residues concentrated in the CTD, and negatively charged residues dominating in the NTD (Fig. 4C and Supplementary Fig. 5). We assume that these external charges located near the polyene chain have a pronounced impact on the PA, even at a substantial distance to the actual protonation site due to polarization of the conjugated system. To test this hypothesis, we performed PA calculations for ECN in several model potentials (see Supplementary information) based on the OCP structure. Notably, the consideration of the local electric field within the protein in our calculations increased the relative PA of ECN and diminished the required proton transfer energy (Supplementary Table 6). According to our estimation, the chloride ion, which can be present in the OCP^WW^ structure (Fig. 2), provides an additional reduction of the PA difference, which might promote proton transfer. Interestingly, similar to the first WT OCP structure^62^, we observe a chloride ion in the interdomain cavity in two of the three structures we obtained for OCP^WW^ (Fig. 2A, B). In spite of the recurrent presence of the chloride ion in OCP crystals and its proximity to the carotenoid molecule, its functional role has not been elucidated yet. The chloride ion is located approximately 14 Å away from both Tyr-201 and Trp-288, but only 4 Å away from the C12 atom of ECN, reducing the electrostatic potential along the ECN conjugated system, especially in the CTD (see Supplementary Fig. 4). Additionally, we tested whether pH affects OCP photoactivity. We found that the pH value affects the charge distribution in the protein by changing the protonation state of charged amino acids; however, the relative PA remains unchanged (see Supplementary Table 7 and Supplementary Fig. 6). Theoretical results suggest that a change in solution pH would not change the efficiency of the photoinduced proton transfer in OCP. In an additional in vitro experiment, we show that WT OCP is photoactive even at extremely low (3) and very high (11) pH (see Supplementary Fig. 7), although the protein stability is limited to a pH 4-10 range.

We assume that the disruption of the hydrogen bond via Reaction Pathway B is facilitated by the asymmetric protein environment due to its impact on the local electric field. Apparently, such a local charge distribution must also interfere with the carotenoid’s excited charge transfer states. The rise of an ICT state requires extensive mixing of the lowest-lying 1^1^B_u_-like ionic and 2^1^A_g_-like covalent states (see Mixed State Figs. 3 and 4), thus the ICT state is a charge transfer (ionic-like) state with extensive (covalent-like) bond order reversal and a very large (~25 Debye) dipole moment^63^. Thus, photoexcitation of ECN in OCP, leading to the accumulation of the ICT state, results in a shift of electron density towards the keto oxygen, thereby increasing its negative charge. We suggest that, promoted by the electric field of the protein environment, this effect causes ionization of one of the hydrogen bond donors and favors excited state proton transfer (ESPT) towards the ECN, with transient formation of a highly unstable oxocarbenium ion.

**Fig. 5:**
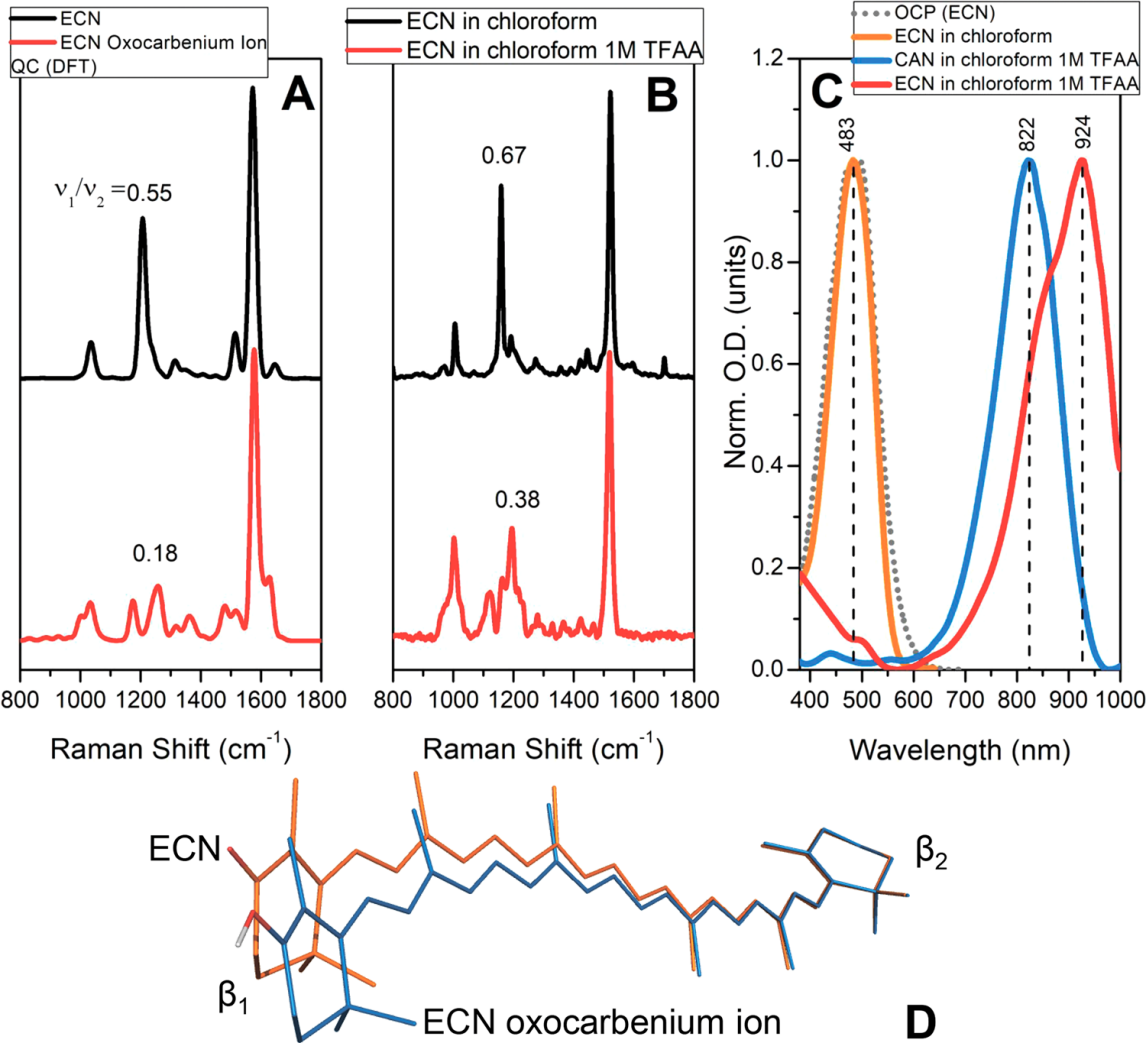
Spectral signatures of protonated carotenoids. Vibrational properties of ECN (black) and the protonated ECN oxocarbenium ion (red), predicted by QC calculations (**A**) and confirmed by steady-state Raman spectroscopy (**B**). **C** Normalized steady-state absorption of ECN (red) and CAN (blue) oxocarbenium ions obtained by protonation of carotenoids using 1 M trifluoroacetic acid (TFAA) in chloroform. For comparison, the absorption of ECN in chloroform (orange) and of OCP in PBS solution (grey dotted line) are presented. **D** Equilibrium geometry of ECN (orange) and the corresponding oxocarbenium ion (blue) according to QC calculations. Structures were aligned at the β_2_-rings.

To directly assess the optical properties of protonated ECN, we performed QC calculations and model experiments (Fig. 5A, B). To measure steady-state absorption and Raman spectra of protonated ECN and CAN, pure carotenoids were dissolved in chloroform with a strong organic acid (1 M trifluoroacetic acid, TFAA) as described in^64^. In polar solvents, both carotenoids are characterized by the S_0_–S_2_ absorption maximum at ~480 nm; however, protonation by TFAA dramatically decreases the energy of this transition as seen from the shifts of the absorption spectra by 350 and 440 nm, respectively, into the near-infrared region, as well as a large reduction of the ratio of ν_2_/ν_1_ Raman bands (C–C and C=C stretching modes, Fig. 5). These effects are explained by a redistribution of electron density, which in turn affects the bond length alternation (BLA) pattern^41^. We would like to note that all effects in our model experiments, including the relative reduction of C–C stretching intensity and the red shift of S_0_-S_2_ absorption (Fig. 5A–C), were completely predicted by our QC calculations with excellent accuracy.

Thus, the accumulation of protonated ECN (the oxocarbenium ion) upon photoexcitation of OCP should be accompanied by an increase of absorption in the IR region (800–1000 nm, see Figs. 3 and 5), considering that formation of the ground state occurs faster than ECN deprotonation. We assume that such signatures of a protonated carotenoid could be identified in IR transient absorption or fs-Raman experiments. However, it is possible that rapid deprotonation occurs within the excited state of ECN. Due to the high proton affinity of Trp and Tyr (see Supplementary Table 6), protonation of ECN must be readily reversible, which might explain the overall low yield of the primary photoproduct (1.5% according to ref. ^9^) upon photoactivation of WT OCP. However, we note that the equilibrium geometry of a protonated carotenoid is different compared to that of ECN in the ground state (Fig. 5D). If deprotonation occurs after conformational relaxation of the oxocarbenium ion, the resulting configuration of ECN could be distorted (and likely not suitable for the formation of hydrogen bonds), which would promote carotenoid isomerization and the following stages of OCP activation.

## Conclusions

The existence of hydrogen bonds between the keto oxygen of the carotenoid and Tyr-201/Trp-288 was revealed by structural studies^2^ long before photoactivity and the functional role of OCP were established^3,17^. Since then, multiple works have proposed the significance of Tyr-201/Trp-288 as hydrogen bond donors for the functional activity of OCP, or rather for its low quantum yield of photoconversion, since these residues are necessary to keep the protein in a compact and physiologically inactive state. Despite the high evolutionary conservation of these residues, we found that both of them do not need to be in place simultaneously to keep the protein photoactive. Our work shows that the presence of two hydrogen bonds in WT OCP causes spectral heterogeneity. However, the complications entailed by this heterogeneity could be overcome by protein modifications which reduce the effective number of hydrogen bonds, but still, keep the protein stable in its dark-adapted state. By this artifice, we obtained an OCP variant (OCP^WW^) with the lowest contribution of the red state reported so far, and, consequently, a beneficially reduced spectral heterogeneity in the dark-adapted state (Fig. 1). Concomitantly, the OCP^WW^ variant proved to have an increased quantum yield of specific carotenoid states which appear on a picosecond timescale upon OCP photoexcitation (Fig. 3). In particular, the yield of S* features in OCP^WW^ was at least 5 times larger compared to WT OCP, which permitted quantitative analysis of the excited state evolution (Fig. 3) and revealed an interplay between the charge transfer (ICT) states and long-lived products (such as S*) of the photochemical reaction. Considering various possible mechanisms for hydrogen bond breaking, we put forward the hypothesis that electronic excitation of the ketocarotenoid in OCP induces accumulation of an ICT state, the features of which—together with the local electric field provided by the specific protein environment within the carotenoid tunnel—promote redistribution of the electron density in a way that it induces proton transfer from Tyr-201 or Trp-288 to the keto oxygen of the carotenoid leading to the formation of a metastable oxocarbenium ion (see Figs. 4 and 5). We assume that the chloride ion, observed in the interdomain cavity of OCP in several crystal structures, might promote transient protonation of the carotenoid as it contributes to the local electrostatic potential. The features of protonated carotenoids, as characterized here by absorption and Raman spectroscopy and supported by quantum chemical calculations, reveal structural determinants forcing the carotenoid to leave its initial position. Since the equilibrium conformation of the ketocarotenoid and its oxocarbenium ion are drastically different, transient protonation creates the driving force for structural changes of the carotenoid’s geometry. We assume that the relaxation of the oxocarbenium ion’s conformation might occur faster than its inevitable deprotonation, resulting in a distorted geometry of the ketocarotenoid lacking hydrogen bonds with Tyr-201 and Trp-288. The protein with the distorted carotenoid might probably reach the point of no return more easily and evolve into the red physiologically active state more efficiently.

Our results indicate clearly that the inherent problem of the low quantum yield of the primary photoproducts in OCP can be solved. This is the prerequisite for acquiring useful information in time-resolved structural experiments at the current scientific frontier with the use of the X-ray Free Electron Lasers. This will be required to unravel the nature of structural/photochemical transformations during the first 20 picoseconds after OCP photoexcitation.

## Methods

### Cloning, protein expression, and purification

Production of wild-type *Synechocystis* OCP and variants thereof in ECN/CAN-producing strains of *Escherichia coli* followed previously published protocols^51^. All OCP variants were produced by site-directed mutagenesis on the basis of the pQE81-L plasmid for the wild-type OCP using Q5 High-Fidelity DNA polymerase (New England Biolabs, USA) and primers indicated in Supplementary Table 8. The integrity and correctness of the resulting constructs were verified by DNA sequencing. The proteins carried an N-terminal His-tag (MRGSHHHHHHTDPATM…) and were purified using a combination of immobilized metal-affinity and size-exclusion chromatography as described before^10^. Protein concentrations were determined by spectrophotometry at 280 nm using the sequence-specific extinction coefficients calculated by the ProtParam tool in ExPasy.

### Crystallization and X-ray data collection

OCP^WW^ was crystallized directly after purification without freezing. The sample (12 mg/ml) was dialyzed into a 10 mM Tris-HCl buffer, pH 7.5, containing 10 mM NaCl and subjected to crystallization by sitting drop vapor diffusion using commercial screens. Similar crystals reaching 100 μm in size and belonging to the same space group P3_2_21 grew under conditions with pH 4.6, 5.5, and 6.5 (Supplementary Table 1) and were mounted on nylon loops and flash-frozen in liquid nitrogen using glycerol as cryoprotectant.

X-ray diffraction data (Table 1) were collected at 100 K at the P14 beamline of DESY-Hamburg synchrotron (Petra III, Germany) using Dectris EIGER 16 M detector.

### Crystal structure solution and refinement

Diffraction data were processed using XDS^65^. The structures were solved using molecular replacement in MOLREP^66^ and the PDB ID 4XB5 structure as a search model, yielding one OCP^WW^ molecule per asymmetric unit. Before refinement, the canthaxanthin molecule present in the 4XB5 structure was removed to avoid bias in structure modeling. ECN was then manually built in the omit density in Coot^67^ and used for refinement using REFMAC^68^. The refinement strategy included rigid-body refinement and then restrained refinement using TLS and individual anisotropic B-factors. Occupancies of alternative conformations of some residues including the engineered Trp-201 residue were also addressed to better describe the electron density map. Atomic coordinates and structure factors are deposited with the Protein Data Bank (PDB) under accession numbers indicated in Table 1 and Supplementary Table 1.

### Molecular dynamics simulations

The initial conformations of Trp-201 (“IN” and “OUT” rotamers) of OCP^WW^ were taken from our crystallographic models. The model of the apo-protein and carotenoid molecules were composed using the OPLS-AA forcefield exactly in the same way as it was made earlier for similar simulations^69^. The molecular model of the carotenoid molecule was created using standard OPLS atom types with the diene types for the conjugated chain. The model globules were dissolved in 100 mM NaCl solution (TIP4P water model). The molecular scene was orthorhombic with periodic boundary conditions and an inter-pinacoid distance of 10 nm. The reference temperature was 310 K (Nose-Hoover thermostat) and the reference isotropic pressure was 1 Bar (Parrinello-Rhaman barostat). The integration step was 2 fs and the hydrogen bonds were constrained using LINKS algorithm. At the first stage of the simulation, the backbone atoms and heavy atoms of the carotenoid molecule were restrained in the space and the surrounding solution and amino acids’ side chains were equilibrated for 10 ns. Then, the restrains were removed and the structure of the initially “IN” and “OUT” OCP^WW^ globules was equilibrated in 100 ns simulation (10 ns tails of the trajectories were used for conformation analysis). Two independent simulations were made for each rotamer.

### Size-exclusion spectrochromatography

OCP WT and OCP^WW^ were analyzed by size-exclusion chromatography with full absorbance spectrum detection. Purified protein samples (50 μM) were loaded on a Superdex 200 Increase 5/150 column (GE Healthcare) equilibrated with a filtered and degassed 20 mM Tris-HCl buffer, pH 7.6, containing 150 mM NaCl and operated at a 0.45 ml/min flow rate using a Varian 335/Varian 363 HPLC system (Varian Inc., Melbourne, Australia). During the run, absorbance in the 240–900 nm range with 1-nm steps (4 nm slit width) was recorded with a frequency of 2.5 Hz. The profiles contained the symmetrical peaks whose absorbance spectra corresponding to the peak maximum and to the apparent Mw of 35 kDa are presented as extracted from diode-array detector data using a custom Python-based script. Apparent Mw for the peaks were determined using column calibration with BSA dimer (132 kDa), BSA monomer (66 kDa), ovalbumin (43 kDa), and α-lactalbumin monomer (15 kDa). The calculated Mw for a OCP WT monomer is 34.6 kDa.

### Steady-state absorption measurements

Steady-state absorption spectra and the time-courses of absorbance changes at 550 nm were recorded as described earlier^23^. A blue light-emitting diode (M455L3, Thorlabs, USA), with a maximum emission at 455 nm was used for the photoconversion of the samples (actinic light for OCP^O^ → OCP^R^ photoconversion). The temperature of the sample was stabilized by a Peltier-controlled cuvette holder Qpod 2e (Quantum Northwest, USA) equipped with a magnetic stirrer. Amplitudes of photoconversion and OCP^R^ → OCP^O^ (R-O for simplicity) relaxation rates were determined according to procedures described earlier^11^ after a 10 s exposure to actinic light at temperatures from 10 to 45 °C. Each experiment was repeated at least three times. Rate constants (k) of a single temperature-dependent process yield a straight line within an Arrhenius plot (${\rm{ln}}{\rm{k}}$ versus T^−1^), from which both the activation energy (*E*_A_) and the pre-exponential factor were determined.

### Transient absorption spectroscopy

Transient absorption spectra were measured using a femtosecond pump-supercontinuum probe setup. The output of Ti:Sapphire oscillator (800 nm, 80 MHz, 80 fs, “Tsunami”, Spectra-Physics, USA) was amplified by a regenerative amplifier (“Spitfire”, Spectra-Physics, USA). The repetition rate of the amplified laser pulses was set at 100 Hz. The amplified pulses (800 nm, 100 Hz, 1.2 mJ, 80 fs) were split into two beams. One of the beams was attenuated to 0.4 mJ and directed to a non-collinear optical parametric amplifier (Clark-MXR), the radiation of which was used as a pump pulse. The pump pulse had a Gaussian pulse shape centered at a wavelength of 520 nm, 26 fs FWHM, and attenuated to 50 nJ pulse energy. The second beam was attenuated to 1 µJ and focused into a 3 mm quartz cell with pure H_2_O to produce a supercontinuum probe pulse. The supercontinuum probe pulse had a smooth spectrum in the wavelength range of 400–900 nm.

The pump and probe pulses were delayed relative to each other by a computer-controlled delay line in the range of 0–500 ps, with a resolution of 3.3 fs to 1 ps. The pulses were then attenuated, recombined, and focused in a sample flow cell with an optical path of 0.5 mm. The pump and probe light spots had diameters of 200 and 80 µm, respectively. The relative polarizations of pump and probe beams were adjusted to 54.7° (the so-called “magic angle”).

The experiments were carried out at 293 K. The circulation rate in the flow cell was 8 ml/min.

The supercontinuum probe signal out of the sample was dispersed by a polychromator («Acton SP-300», Roper Scientific, USA) and detected by a CCD camera («Newton», Andor, USA). Transient absorption spectral changes ΔA(t, λ) were recorded within the range of 400–900 nm.

The measured spectra were corrected for group delay dispersion of the supercontinuum using a procedure described previously^70,71^. Global analysis fitting was performed for the transient absorption spectra using the Glotaran program. With global analysis, all wavelengths were analyzed simultaneously with a set of common time constants.

The mathematical formulation of the kinetic model of OCP^WW^ excited states was reduced to a system of five linear differential equations:

$${\dot{{\rm{X}}}}_{2}=-{{\rm{k}}}_{2}{{\rm{X}}}_{2}$$

$${\dot{{\rm{X}}}}_{{\rm{H}}}={{\rm{k}}}_{2}{{\rm{X}}}_{2}-({{\rm{k}}}_{1}+{{\rm{k}}}_{{\rm{ICT}}}+{{\rm{k}}}_{{\rm{S}}\ast }){{\rm{X}}}_{{\rm{H}}}$$

$${\dot{{\rm{X}}}}_{1}={{\rm{k}}}_{1}{{\rm{X}}}_{{\rm{H}}}-{{\rm{m}}}_{1}{{\rm{X}}}_{1}$$

$${\dot{{\rm{X}}}}_{{\rm{ICT}}}={{\rm{k}}}_{{\rm{ICT}}}{{\rm{X}}}_{{\rm{H}}}-{{\rm{m}}}_{{\rm{ICT}}}{{\rm{X}}}_{{\rm{ICT}}}$$

1 $${\dot{{\rm{X}}}}_{{\rm{S}}\ast }={{\rm{k}}}_{{\rm{S}}\ast }{{\rm{X}}}_{{\rm{H}}}-{{\rm{m}}}_{{\rm{S}}\ast }{{\rm{X}}}_{{\rm{S}}\ast }$$

here X_2_ denote probability of the S_2_ excited state, which decays into the intermediate state S_H_ (denoted by ${{\rm{X}}}_{{\rm{H}}}$) with the rate constant ${{\rm{k}}}_{2}$. S_H_ can decay into any of the S_1_ excited, ICT or S* states with the rate constants ${{\rm{k}}}_{1}$, ${{\rm{k}}}_{{\rm{ICT}}}$, and ${{\rm{k}}}_{{\rm{S}}\ast }$, respectively. The latter three states decay into the ground S_0_ state with the rate constants ${{\rm{m}}}_{1}$, ${{\rm{m}}}_{{\rm{ICT}}}$, and ${{\rm{m}}}_{{\rm{S}}\ast }$, respectively. The sum of the probabilities is conserved during the dynamics:

$${{\rm{X}}}_{2}+{{\rm{X}}}_{{\rm{H}}}+{{\rm{X}}}_{1}+{{\rm{X}}}_{{\rm{ICT}}}+{{\rm{X}}}_{{\rm{S}}\ast }+{{\rm{X}}}_{{\rm{S}}0}=1$$

The characteristic polynomial of the system (1) has five nonzero roots (eigenvalues), which represent five characteristic times of electronic transitions. The eigenvalues were found numerically by solving the characteristic polynomial and the general solution of the system of linear equations by inverting the corresponding kinetic matrix. A particular solution was found by choosing X_2_(0) = 1 and zero for the other variables. The kinetic parameters were found by numeric nonlinear minimization of the solution to fit the experimental measurements in the spectral interval of 400–910 nm and the time range of 0.05–200 ps. The relative contributions of different channels were calculated by the rate constants ${{\rm{k}}}_{1}$, ${{\rm{k}}}_{{\rm{ICT}}}$, and ${{\rm{k}}}_{{\rm{S}}\ast }$, respectively.

### Quantum chemistry

In the present work, the following DFT functionals were used for geometry optimization: B3LYP^72^, CAM-B3LYP^73^, PBE0^74^. Dispersion correction D3^74^ was used in all cases. Basis sets used were: 6–311 G**/6-311++**^75,76^, def2-TZVPP^77^. To calculate the interaction energy in H-bonded complexes, the BSSE approach was used^78^. For proton affinity calculations, a direct Δ approach via the energy difference between two calculations of the initial and final states was used^79^. To simulate the electrostatic surrounding, point charges were introduced to the quantum chemical system. For S_2_ calculations, TDDFT/TDA/B3LYP, CAM-B3LYP^73^, wB97X^74,80^, RI-wB2PLYP^81^ functionals were used. These calculations were done using ORCA 4.2 software package.

### Quantum chemical procedure for S_1_ state calculation

Excitation energies of ECN were calculated by CASSCF method with perturbation theory refinement using RI-XMCQDPT2 method. Five states were included in state averaging: the ground ($1{{\rm{A}}}_{{\rm{g}}}^{-}$), the bright ($1{{\rm{B}}}_{{\rm{u}}}^{+}$) and three dark states (($2{{\rm{A}}}_{{\rm{g}}}^{-}$, $3{{\rm{A}}}_{{\rm{g}}}^{-}$, $1{{\rm{B}}}_{{\rm{u}}}^{-}$). The active space in CASSCF included 10 orbitals and 10 electrons. MP2-natural orbitals were taken as an initial guess. The Def2-SVP atomic basis set was used. Calculations were performed using the Firefly QC package, which is partially based on the GAMESS (US) source code^82^.

### Statistics and reproducibility

Student’s t-test and analysis of variance (ANOVA) were performed to compare the mean of the control group to the mean of the treatment group. *p* < 0.05 was considered significant. Normality of distribution was examined by using Shapiro–Wilk normality test. The data were considered as normally distributed if *p* > 0.05. Equality of variance was examined with an F test. *p* > 0.05 was considered as equal variances. The data shown are mean values of three independent experiments with error bars corresponding to standard errors. All spectroscopic experiments were repeated at least 3 times for independently prepared samples. Upon sample preparation, the purity was checked by standard biochemical approaches (SDS-PAGE, SEC, and characterization by absorption spectroscopy). The data collected in independent experiments exhibit uniform properties, thus providing evidence of a sufficient sample size. All statistical analysis was performed by using OriginPro 2015 (OriginLab Corp., Northampton, MA, USA).

### Reporting summary

Further information on research design is available in the Nature Research Reporting Summary linked to this article.

## Supplementary information

Supplementary Information

Description of Additional Supplementary Files

Supplementary Data 1

Supplementary Data 2

Supplementary Data 3

Supplementary Data 4

Reporting Summary

## Data Availability

The refined models and structure factor amplitudes have been deposited in the PDB with the following accession codes 6T6K, 6T6M, and 6T6O. Figures 1, 2, 3, and 4 have associated raw data. Other data will be made available to any reader directly upon request.
