# Supplementary material for: Role of hydrogen bond alternation and charge transfer states in photoactivation of the Orange Carotenoid Protein
Source: Commun Biol. 2021 May 10;4:539. doi: 10.1038/s42003-021-02022-3 (PMC8110590; doi:10.1038/s42003-021-02022-3)
Supplement: Supplementary file 1 — Supplementary Information [file 42003_2021_2022_MOESM1_ESM.pdf]

SUPPLEMENTARY INFORMATION for

Role of hydrogen bond alternation and charge transfer states in photoactivation of the Orange Carotenoid Protein

Igor A. Yaroshevich[1], Eugene G. Maksimov[1,2*], Nikolai N. Sluchanko[2], Dmitry V. Zlenko[1], Alexey V. Stepanov[3], Ekaterina A. Slutskaya[1,3], Yury B. Slonimskiy[1,2], Viacheslav S. Botnarevskii[1,2], Alina Remeeva[4], Ivan Gushchin[4], Kirill Kovalev[4,5,6,7,8], Valentin I. Gordeliy[4,5,6,7], Ivan V. Shelaev[9], Fedor E. Gostev[9], Dmitry Khakhulin[10], Vladimir V. Poddubnyy[11], Timofey S. Gostev[1], Dmitry A. Cherepanov[9,12], Tomáš Polívka[13], Miroslav Kloz[14], Thomas Friedrich[15], Vladimir Z. Paschenko [1], Victor A. Nadtochenko[9], Andrew B. Rubin[1], Mikhail P. Kirpichnikov[1,3]

1 Faculty of Biology, M.V. Lomonosov Moscow State University, 119992, Moscow, Russia

2 A.N. Bach Institute of Biochemistry, Federal Research Center of Biotechnology of the Russian Academy of Sciences, Moscow, Russia

3 M.M. Shemyakin and Yu.A. Ovchinnikov Institute of Bioorganic Chemistry, Russian Academy of Sciences, Miklukho-Maklaya Str., 16/10, Moscow, 117997, Russia

4 Research Center for Molecular Mechanisms of Aging and Age-Related Diseases, Moscow Institute of Physics and Technology, 141700 Dolgoprudny, Russia

5 Institut de Biologie Structurale J.-P. Ebel, Université Grenoble Alpes-CEA-CNRS, 38000 Grenoble, France

6 Institute of Biological Information Processing (IBI-7: Structural Biochemistry), Forschungszentrum Jülich, 52428 Jülich, Germany

7 JuStruct: Jülich Center for Structural Biology, Forschungszentrum Jülich, 52428 Jülich, Germany

8 Institute of Crystallography, RWTH Aachen University, 52062 Aachen, Germany

9 N.N. Semenov Federal Research Center for Chemical Physics, Russian Academy of Sciences, Russia, Kosygina st., 4, 119991 Moscow, Russia

10 European XFEL GmbH, Holzkoppel 4, 22869 Schenefeld, Germany

11 Chemistry department, M.V. Lomonosov Moscow State University, 119992, Moscow, Russia

12 A.N. Belozersky Institute of Physical-Chemical Biology, Moscow State University, 119992 Moscow, Russia,

13 Institute of Physics, Faculty of Science, University of South Bohemia, Branišovská 1760, 37005 České Budějovice, Czech Republic

14 ELI-Beamlines, Institute of Physics, Na Slovance 2, 182 21 Praha 8, Czech Republic

15 Technische Universität Berlin, Institute of Chemistry PC14, Straße des 17. Juni 135, 10623 Berlin, Germany

* e-mail: emaksimoff@yandex.ru

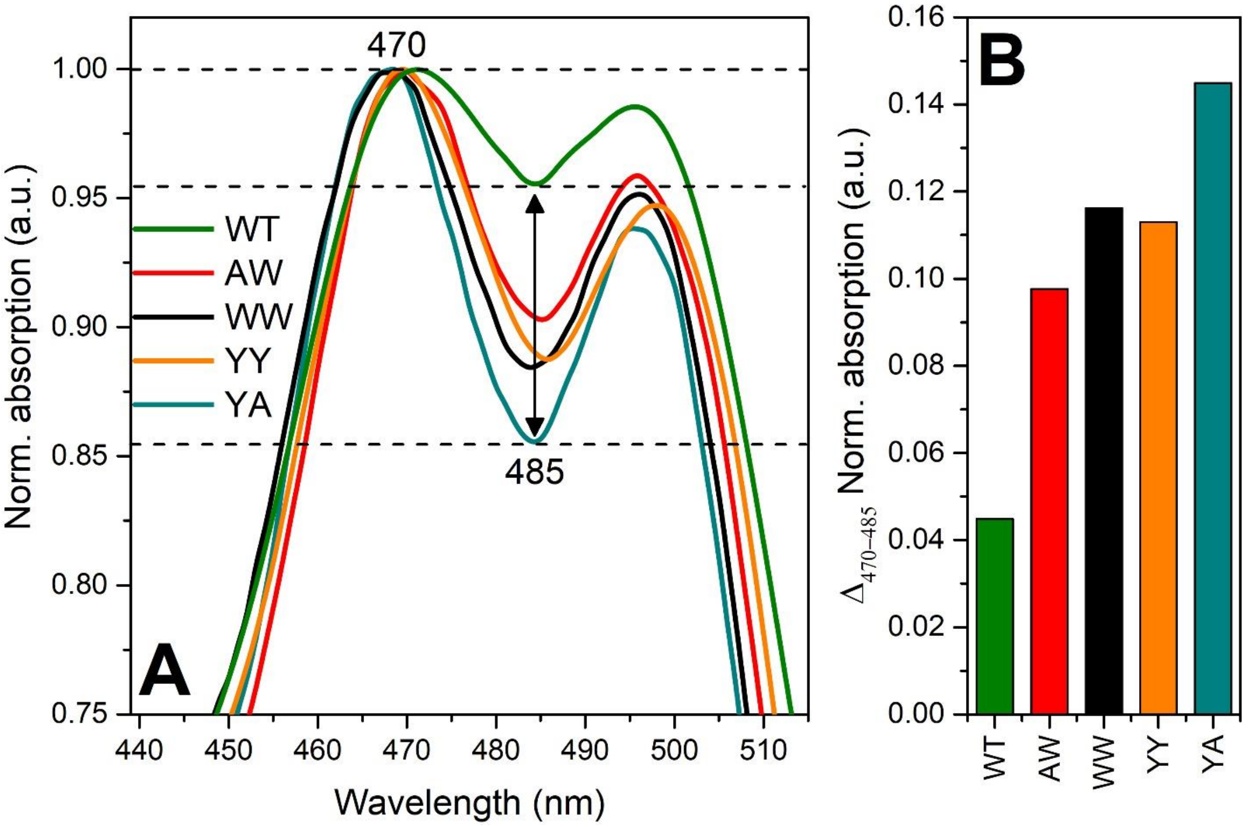

Supplementary Figure 1. (**A**) – normalized absorption spectra of orange fractions of OCP variants from Figure 1 after spectral decomposition and subtraction of absorption of red (OCP^{WT}, OCP^{WW}) or purple (OCP^{YA}, OCP^{YY}, OCP^{AW}) states. (**B**) – relative difference in absorption intensity at local maximum (470 nm) and minimum (485 nm) of species form Supplementary Figure 1A indicating the sharpness of vibrionic structure in different OCP variants.

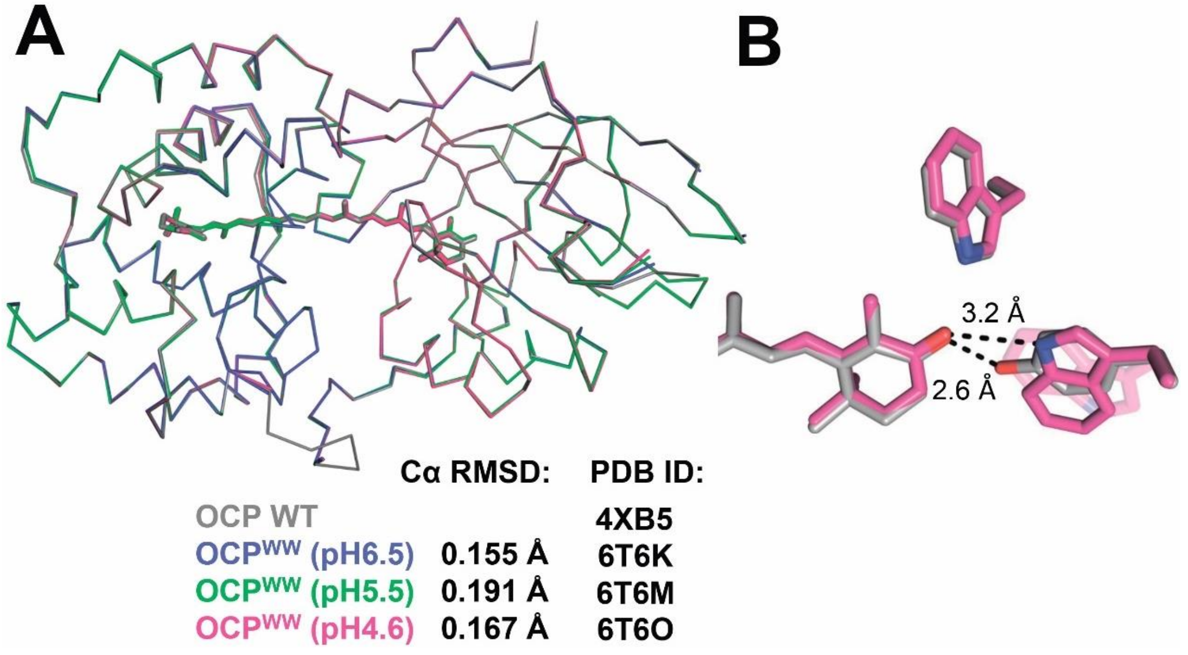

Supplementary Figure 2. (**A**) - overlay of the OCP^{WW} crystal structures obtained at different pH values, with the structure of OCP WT (PDB IDs are indicated along with the Cα RMSD values obtained upon pairwise alignment with the reference WT OCP structure). (**B**) - organization of hydrogen bonds between the keto-oxygen of ECN and Tyr-201 (gray) in WT OCP and Trp-201 (pink) in OCP^{WW} (PDB ID 6T6O).

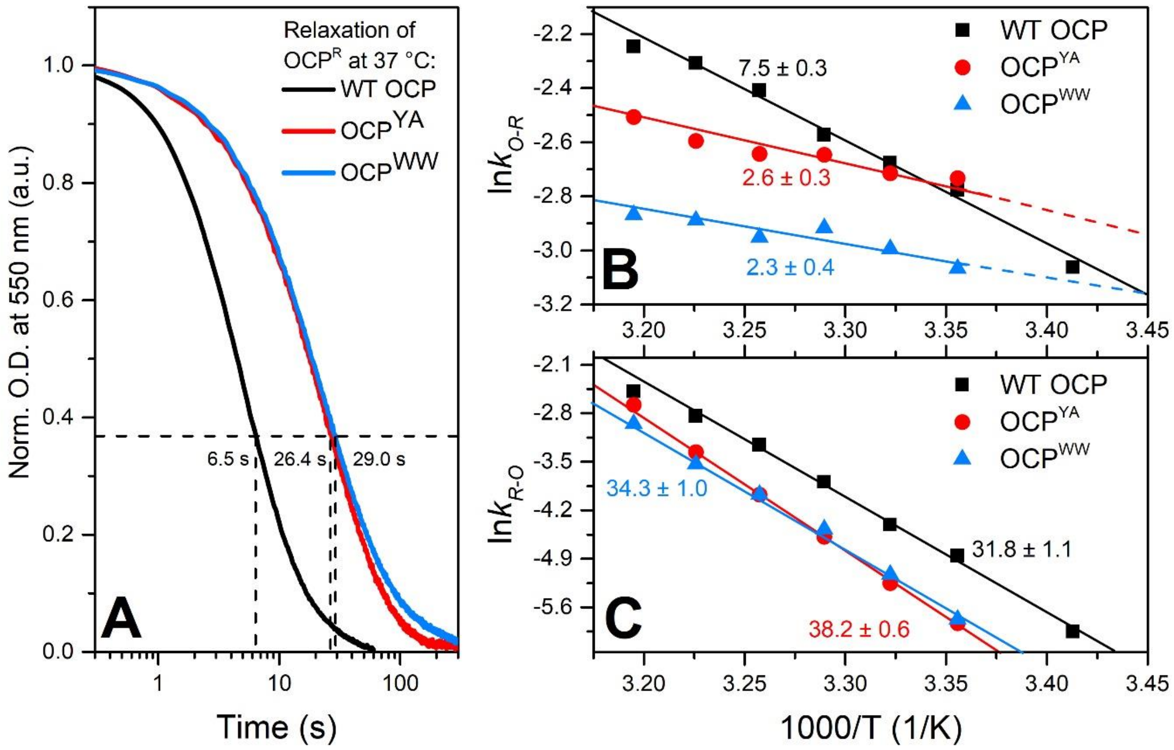

Supplementary Figure 3. (**A**) – time courses of back-conversion of the photoactivated OCP variants at 37 °C. The characteristic time constants are indicated. Arrhenius plots of (**B**) photoactivation (O-R) and (**C**) relaxation (R-O) rates of OCP variants and corresponding energy barriers. N=3 biologically independent experiments.

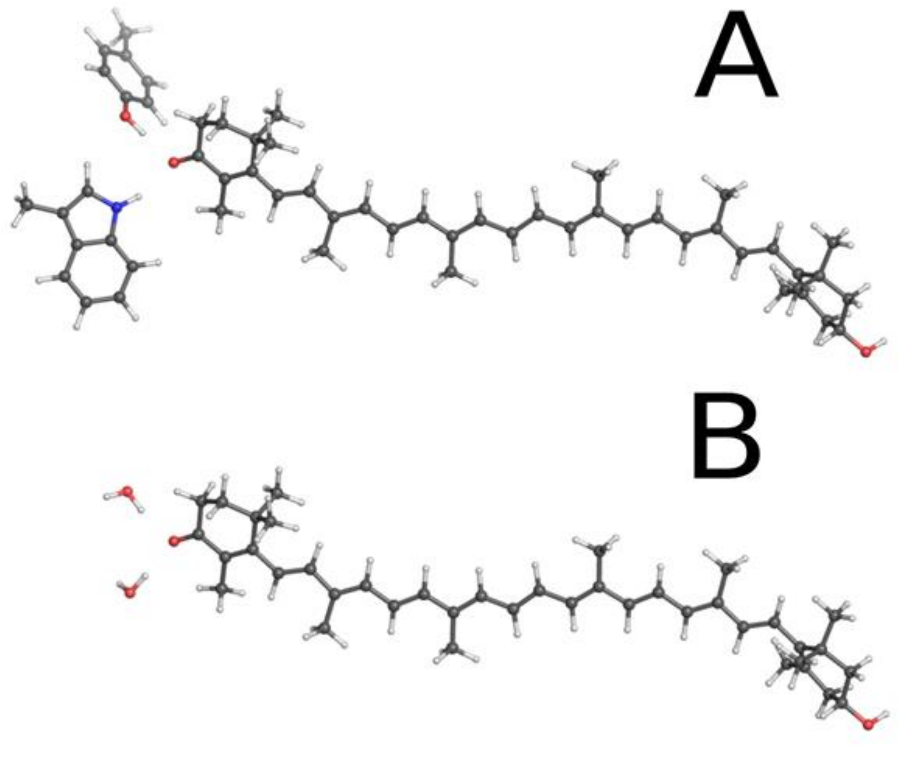

Supplementary Figure 4. ECN/Tyr-201/Trp-288 (**A**) and ECN-2H_2O (**B**) complexes optimized with B3LYP/D3/6-311G++**.

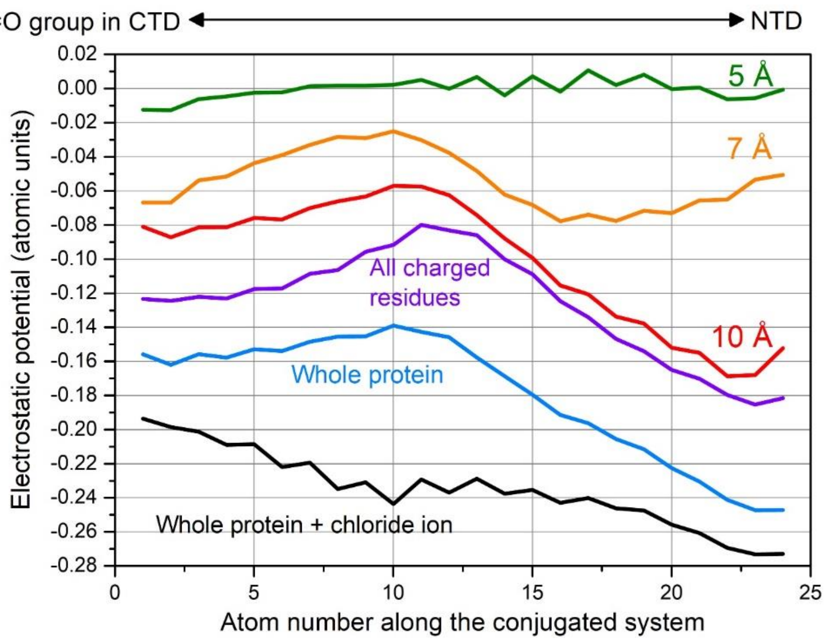

Supplementary Figure 5. The distribution of the external electrostatic potential along the ECN conjugated system positions generated by different protein parts. Residues that are 5, 7 and 10 Å away from a specific ECN carbon atom along the conjugated system were considered (green, orange and red lines, respectively). Further, all charged residues (violet line) and a chloride ion in the interdomain cavity near the ECN (black line) were taken into account. All OCP amino acids generate the electrostatic potential at ECN shown in blue.

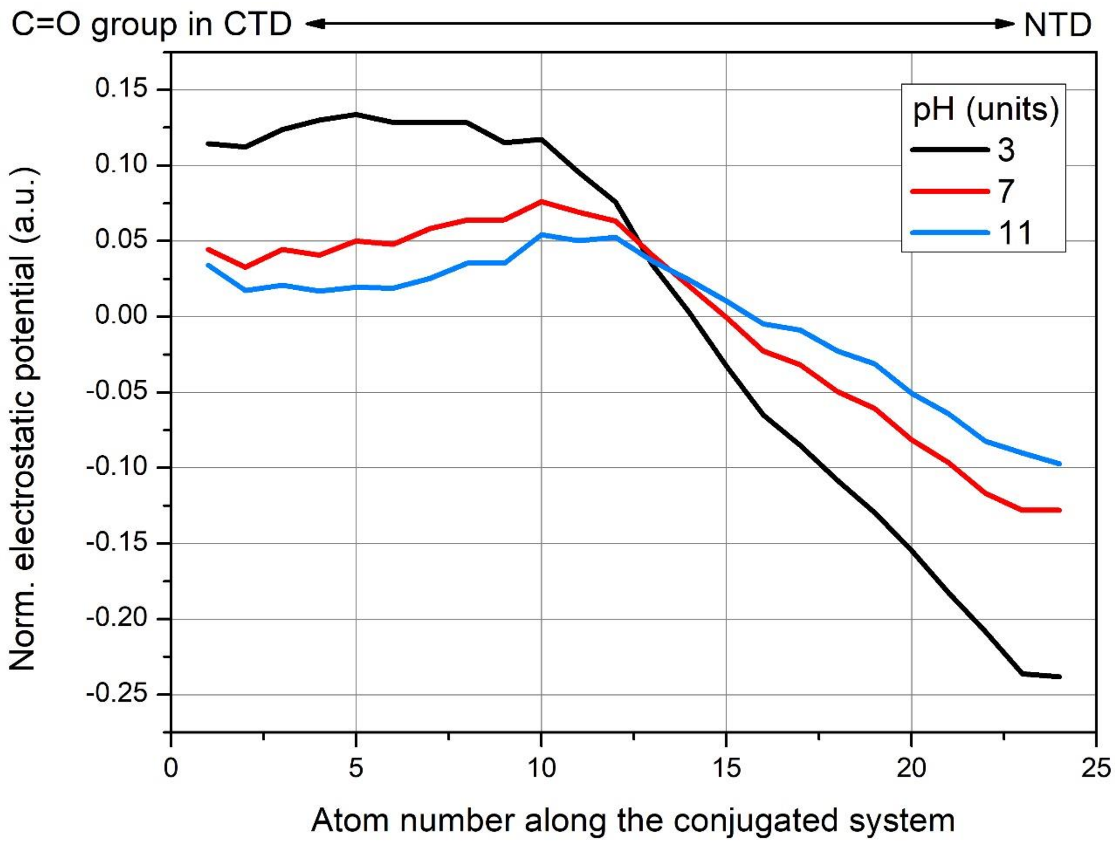

Supplementary Figure 6. The distribution of the external electrostatic potential along the ECN conjugated system positions generated for different pH values: 3 (black), 7 (red), 11 (blue). Average value of electrostatic potential for all atoms in conjugated system of carotenoid was subtracted from each individual point for better presentation.

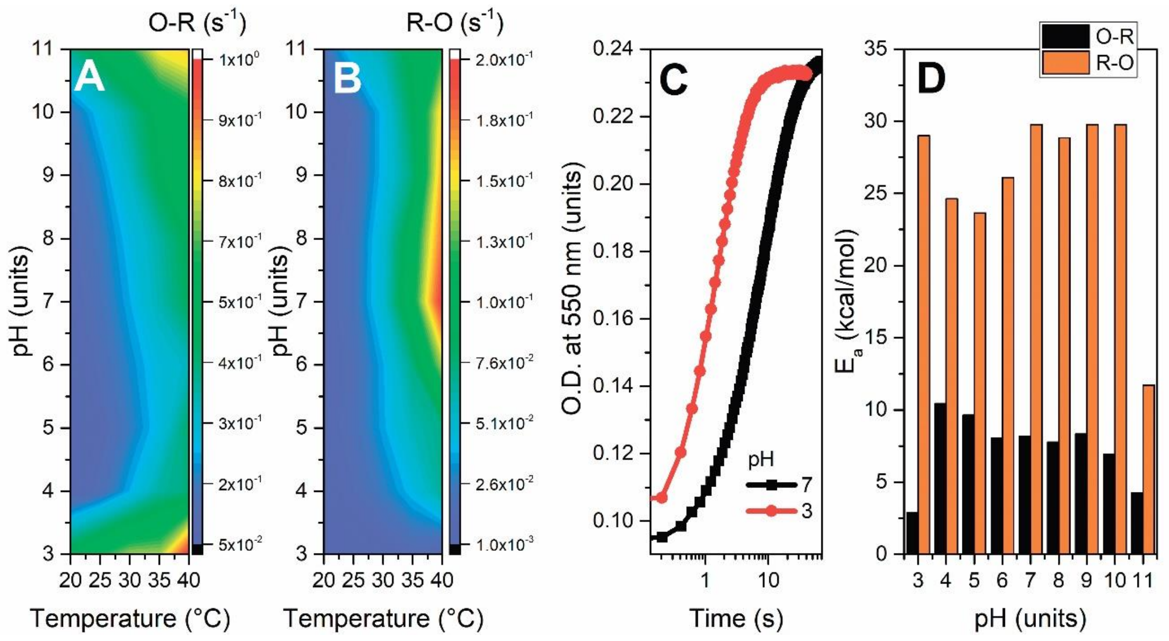

Supplementary Figure 7. Effect of pH on photoactivation of OCP. The apparent rate constant of the OCP^O to OCP^R transition (**A**) under continuous illumination of the WT OCP sample by actinic light (average power of 445 nm LED was set to 200 mW) and following relaxation (**B**) is shown as a function of temperature and pH. The distribution of rate constants is presented as a color-coded image with corresponding legends at the right side of the images. (**C**) – characteristic time courses of O.D. changes at 550 nm of WT OCP under constant actinic illumination at 20 °C measured in a buffer with pH 3 (red) and 7 (black). N=3 biologically independent experiments.

Supplementary Table 1. OCP^{WW} crystallization conditions.

	pH 6.5	pH 5.5	pH 4.6
PDB ID	6T6K	6T6M	6T6O
Conditions	0.1M DL-glutamic acid monohydrate; 0.1M DL-alanine; 0.1M glycine; 0.1M DL-lysine monohydrochloride; 0.1M DL-serine; 0.1M imidazole; MES monohydrate (acid), pH 6.5;<br>20% v/v ethylene glycol; 10 % w/v PEG 8000	0.1 M sodium citrate pH5.5<br>20 % w/v PEG 3000	0.1 M sodium acetate pH4.6<br>8 % w/v PEG 4000
Protein concentration, mg/ml	12	12	12
Temperature, °C	20	20	20

Geometry and energy of hydrogen bonds

The geometries and energies for the H-bonded ECN-Tyr-201/Trp-288 complex were calculated with various DFT functionals listed in Supplementary Tables 2 and 3 below. The energy of the H-bond was calculated with a BSSE approach for the respective molecular complex in vacuo [1].

Supplementary Table 2. Geometry of the equilibrium state of the ECN-Tyr-201/Trp-288 complex calculated with various DFT functionals. Distances between keto oxygen ECN(O) and corresponding hydrogen X(H), or corresponding heavy atom Y(O)/W(N) are listed in Å.

Method	ECN(O)-Y(H), Å	ECN(O)-Y(O), Å	ECN(O)-W(H), Å	ECN(O)-W(N), Å
B3LYP/D3/6-311G++**	1.86	2.82	2.08	3.05
PBE0/6-311G++**	1.78	2.75	1.97	2.94
CAM-B3LYP/D3/6-311G++**	1.82	2.78	2.02	2.98

Supplementary Table 3. Energy of the ECN-Tyr-201/Trp-288 complex calculated with BSSE and various DFT functionals in kcal/mol.

Method	(ECN-W)-Y	(ECN-Y)-W	ECN-YW
B3LYP/D3/6-311G++**	6.25	4	13.25
PBE0/6-311G++**	7.5	5	15
CAM-B3LYP/D3/6-311G++**	7.75	5.25	15.25

The potential energy surfaces for the ECN/Y201/W288 complex were calculated with B3LYP/D3/6-311G++** level of theory and fitted with analytic functions as exemplified in [2], with an addition of the term-dependent dihedral angles $E_{PES}(\theta)$ and $h(D_{Hb})$:

$$E_{Hb} = E_{PES}(\theta) + f(R_{Hb}) + g(A_{Hb}) + h(D_{Hb}),$$

Here, E_{Hb} is an energy of a deformed H-bond; $E_{PES}(\theta)$ the energy of ECN end ring rotation around the C6-C7 bond (obtained with PES calculation as it was done in [70]); $f(R_{Hb})$ is the energy of the H-bond dependent on the distance R_{Hb} between proton donor (O for Tyr or N for Trp) and acceptor atom (keto-O of ECN), that is the only term different for the ECN-Y and ECN-W bond that defines the energy of the minimum for the H-bond energy; $g(A_{Hb})$ represents the energy of the H-bond dependent on the angle A_{Hb} between the proton donor atom, the proton acceptor (the bond to the keto-oxygen at the C4 atom of ECN); $h(D_{Hb})$ is the energy of the H-bond dependent on the dihedral angle D_{Hb} between the proton donor atom, the proton acceptor, and the C4 and C5 atoms of ECN. $f(R_{Hb}), g(A_{Hb}), h(D_{Hb})$ are defined as:

$$f(R_{Hb}) = p_i\left[\left(\frac{R_{0i}}{R_{Hb}}\right)^6 - \left(\frac{R_{0i}}{R_{Hb}}\right)^4\right],$$

$$g(A_{Hb}) = 17.5[1 - \cos(A_{Hb} - 112)],$$

$$h(D_{Hb}) = 4.25[1 - \cos(2D_{Hb})] + 6\cos\left(\frac{D_{Hb}}{2}\right)$$

Parameters of fitting are:

Parameter	ECN-Y	ECN-W
p_i	200	135
R_{0i}	2.29	2.45

To estimate the H-bond energy for the compact OCP state, the steric parameters $\theta, R_{Hb}, A_{Hb}, D_{Hb}$ were obtained from the crystal structures (Supplementary Table 4).

Supplementary Table 4. Parameters of the hydrogen bonds (Hb) obtained from available crystals

	θ	R_{Hb}, Tyr	A_{Hb}, Tyr	D_{Hb}, Tyr	R_{Hb}, Trp	A_{Hb}, Trp	D_{Hb}, Trp
3MG1, cA	146	2.6	109	169	2.9	140	30
3MG1, cB	149	2.6	110	170	2.9	139	32
3MG2, cA	151	2.6	108	175	2.9	143	14
3MG2, cB	155	2.6	109	178	3	141	25
3MG3, cA	141	2.6	108	170	2.9	140	32
3MG3, cB	143	2.6	106	170	2.9	141	32
5UI2, cA	103	2.9	108	159	2.9	139	39
5UI2, cB	87	2.8	112	164	2.9	140	28
5TUX	132	2.6	110	170	2.9	136	32
5TV0	135	2.6	112	168	2.9	141	20
5HGR, cA	109	2.6	103	176	2.9	139	36
5HGR, cB	115	2.6	110	173	2.9	138	37

Energy of hydrogen bond breakage upon excitation

The energy of H-bond breakage in the excited state can be estimated as the sum of the dissociation energy in the ground state and a shift of the excited state energy upon formation of the H-bonded complex [3,4]. The bathochromic shift of the S_2 state upon the ECN-YW H-bonded complex formation indicates an increase of the H-bond energy in this state. Similarly, a strong bathochromic shift of the S_2 state upon oxocarbenium ion (ECN(H+)) formation indicates a substantial rise of the energy corresponding to the interaction between the keto oxygen and the proton. Calculations of the excited state energies for molecular complexes ECN-W, ECN-Y and ECN-YW were performed for equilibrium vacuum geometries described in the previous section.

Supplementary Table 5. Calculated energies for the excitation in free ECN, ECN H-bonded to Tyr-201, to Trp-288 and to both, and for two oxocarbenium ions of ECN.

Compound (Geometry)	S1 (CI), cm^{-1}	S_2 (TDDFT(wB97X/D3/TDA/6-311G**)), cm^{-1}	S_2 (TDDFT(CAMB3LYP/D3/TDA/6-311G**)), cm^{-1}	S_2 (TDDFT(RI-wB2PLYP/def2-TZVPP)), cm^{-1}
ECN	20210	22533.8	21322.4	23812.8
ECN-W	19723	21766.0	20498.9	22983.6
ECN-Y	19574	21721.3	20443.2	22937.1
ECN-YW	19540	21365.8	20004.8	22528.3
ECN(H+)	-	14238.7	14399.9	13420.3
ECN(2H+)	-	16645.9	16269.5	16129.6

The energy of the H-bond breakage upon charge separation mechanism is estimated as a difference in the proton affinity (ΔPA) of the proton donor (Trp or Tyr) and acceptor (ECN) in the corresponding ground electronic states (Supplementary Scheme 1) [5,6].

1) $\mathbf{ECN + Tyr(H)/Trp(H) \longrightarrow ECN(H)^{+} + Tyr^{-}/Trp^{-}} + \Delta PA$

2) $\mathbf{ECN + H^{+} \longrightarrow ECN(H)^{+}} + PA_{ECN}$

3) $\mathbf{Tyr(H)/Trp(H) \longrightarrow Tyr^{-}/Trp^{-} + H^{+}} - PA_{AA}$

Supplementary Scheme 1. The reaction of the proton transfer in the ECN-Tyr-201/Trp-288 complex. Note that in reactions 1 and 3 only one proton is taken away from the donating residue, which might be either Trp or Tyr. PA_{ECN} and PA_{AA} represent the absolute proton affinity of the carotenoid and amino acid, respectively. ΔPA is an energetic difference between reagents and products calculated as ($PA_{ECN} – PA_{AA}$).

In the vacuum, the ΔPA is high (see relative value in Supplementary Table 6 for vacuum compounds) even in relation to the excitation energy, so the proton transfer is improbable due to the energy deficiency. However, the effect of the protein environment might be to significantly shift the energetic balance. Therefore, calculations taking the electrostatics of the environment into account were also performed. These calculation results are presented in Supplementary Table 6.

Since carotenoids are hydrophobic molecules, it is expected that the high-affinity binding pocket of the carotenoid-binding site should as well be hydrophobic (i.e. lacking charged groups). On the other hand, OCP is a soluble protein, so it should contain a substantial amount of charged groups. It is quite interesting to sort out, how these seemingly opposite traits are combined within a rather small protein. To estimate the charge distribution in OCP and its impact on the energy of the proton transfer we used the crystal structure (PDB ID 4XB5) and parameterized it with a partial charge formalism using the CHARMM force field [7] (Supplementary Figure 5). Then, in the presence of frozen point charges, which simulates the charge distribution in the protein, the geometries of ECN and Tyr-Trp complex were optimized using

B3LYP/D3/6-311G**/6-311++**. Several constraints were used: the position of the proton donor and acceptor atoms, the position of the peptide groups for the Tyr-Trp complex and the position of the center of mass for ECN. Then, optimized geometries were used to calculate PA values in the presence of the protein's electrostatic potential with four sets of the accounted point charges: "only charged AA" - point charges corresponded only to the atoms of the charged amino acids; "all AA" - point charges corresponded to all protein atoms; and two modifications of the previous sets which take into account the chloride anion in the corresponding position relative to the ECN as obtained from the OCP^{WW} crystal structure - "only charged AA + Cl^-" and "all AA + Cl^-".

Supplementary Table 6. Calculated PA for the compounds from ECN-YW hydrogen bonded complex.

Compound	Proton affinity, Absolute value ($PA_{compaund}$), kcal/mol			ΔPA (PA_{ECN} – $PA_{Tyr-Trp}$), kcal/mol		
Vacuum						
	B3LYP/D3/6-311G++**	CAM-B3LYP/D3/6-311G++**	PBE0/D3/6-311G++**	B3LYP/D3/6-311G++**	CAM-B3LYP/D3/6-311G++**	PBE0/D3/6-311G++**
ECN	253	244	252			
Tyr-Trp complex	329	329	328	76	85	76
ESP (only charged AA)						
ECN	339	331	338	71	79	71
Tyr-Trp complex	410	410	409			
ESP (only charged AA + Cl^-)						
ECN	368	360	367	62	70	62
Tyr-Trp complex	430	430	429			
ESP (all AA)						
ECN	362	354	361	-15	-8	-17
Tyr-Trp complex	346	346	345			
ESP (all AA + Cl^-)						
ECN	395	386	394	-28	-21	-29
Tyr-Trp complex	366	366	364			

The gradient of the electrostatic field, which occurs in the carotenoid-binding cavity, is generated by groups that are 7-10 Å distant from the carotenoid.

Effect of pH on the energy of the proton transfer

The pH value affects the charge distribution in the protein by changing the protonation state of charged amino acids. To test, whether pH can significantly change the estimated energy of proton transfer, two additional sets of point charges were used. Both sets were calculated with PDB2PQR tool [https://academic.oup.com/nar/article/35/suppl_2/W522/2920806?login=true] using the CHARMM force field for pH 3 and 11. The total charge of the protein changes from 20 for pH 3 to -17 for pH 11 which causes a significant shift of the electrostatic potential baseline, but the electrostatics gradient pattern and relative PA values remained unchanged (Supplementary Table 7, Supplementary Figure 6). These theoretical results suggest that a change in solution pH would not significantly change the efficiency of the photoinduced proton transfer in OCP.

Supplementary Table 7. Calculated PA values for the compounds of the ECN-YW hydrogen bonded complex for different pH values

Compound	Proton affinity, Absolute value ($PA_{compaund}$), kcal/mol			ΔPA ($PA_{ECN} – PA_{Tyr-Trp}$), kcal/mol		
pH 7						
	B3LYP/D3/6-311G++**	CAM-B3LYP/D3/6-311G++**	PBE0/D3/6-311G++**	B3LYP/D3/6-311G++**	CAM-B3LYP/D3/6-311G++**	PBE0/D3/6-311G++**
ECN	362	354	361	-15	-8	-17
Tyr-Trp complex	346	346	345			
pH 3						
ECN	-78	-78	-76	-38	-39	-42
Tyr-Trp complex	-116	-117	-118			
pH 11						
ECN	513	503	512	-28	-19	-29
Tyr-Trp complex	485	484	483			

In an additional in vitro experiment, we found out that WT OCP is photoactive even at very low (3) and very high (11) pH, when it is in the dark-adapted orange state. Upon exposure to actinic blue light (LED, 445 nm, 200 mW), WT OCP converted into the red state; however, the rate constant of this process was pH dependent (Supplementary Figure 7A). The rate constant of the OCP^R to OCP^O relaxation was considered in order to estimate the rate of accumulation of the photoproduct (O-R), since faster relaxation leads to sooner formation of the equilibrium (see Maksimov, E.G., et al., The Signaling State of Orange Carotenoid Protein. Biophys. J., 2015. **109**(3): p. 595-607.). In general, the O-R rate constant increased slightly upon an increase of pH from 4 to 10. However, at pH 3 and pH 11, the O-R transition was dramatically accelerated (Supplementary Figure 7). The apparent activation energy of WT OCP photoactivation was decreased at extremely low and extremely high pH values. Therefore, we conclude that OCP might be photoactivated in a wide range of pH values (4-10). However, we would like to avoid speculations regarding a faster and easier accumulation of the final red OCP state at low (or high) pH, since this phenomenon might be related to a destabilization of protein matrix by conditions far from optimum pH. Indeed, at pH 3 and pH 11 we observed a gradual increase of light scattering upon photoactivation indicating that protein aggregates were formed under such conditions. We would also like to note that in the dark-adapted orange form of OCP, the keto group of the carotenoid and the Tyr-201 and Trp-288 residues are largely shielded from interaction with the solvent. For this reason, we suggest that the pH effects observed in the experiment are associated with the recharging of the amino acid residues at the outer surface of the protein.

Supplementary Table 8. Primers used for site-directed mutagenesis

Primer	Sequence
OCP Y201A Forward	CGTGCTGAACGCAATGGATAATCTGA
OCP Y201A Reverse	TCAGATTATCCATTGCGTTCAGCACG
OCP Y201W Forward	CGT GCT GAA CTG GAT GGA TAA TCT GA
OCP Y201W Reverse	TCA GAT TAT CCA TCC AGT TCA GCA CG
OCP W288Y Forward	GAA TAT TGC ATA TCG TTT TCT G
OCP W288Y Reverse	CAG AAA ACG ATA TGC AAT ATT C
OCP W288A Forward	GAA TAT TGC ATG GCG TTT TCT G
OCP W288A Reverse	CAG AAA ACG CCA TGC AAT ATT C
OCP EcoRI Forward	TCACACAGAATTCATTAAAGAGGAG
OCP after TAA Reverse	CTTGGCTGCAGGTCG

Supplementary References

1 Gutowski, M. & Chal/asiński, G. Critical evaluation of some computational approaches to the problem of basis set superposition error. *The Journal of Chemical Physics* **98**, 5540-5554, doi:10.1063/1.464901 (1993).

2 Fabiola, F., Bertram, R., Korostelev, A. & Chapman, M. S. An improved hydrogen bond potential: Impact on medium resolution protein structures. *Protein Sci.* **11**, 1415-1423, doi:10.1110/ps.4890102 (2002).

3 Zhao, G.-J. & Han, K.-L. Hydrogen Bonding in the Electronic Excited State. *Accounts of Chemical Research* **45**, 404-413, doi:10.1021/ar200135h (2012).

4 Song, P. & Ma, F.-C. Intermolecular hydrogen-bonding effects on photophysics and photochemistry. *International Reviews in Physical Chemistry* **32**, 589-609, doi:10.1080/0144235X.2013.811891 (2013).

5 Moser, A., Range, K. & York, D. M. Accurate Proton Affinity and Gas-Phase Basicity Values for Molecules Important in Biocatalysis. *The Journal of Physical Chemistry B* **114**, 13911-13921, doi:10.1021/jp107450n (2010).

6 Abi, T. G., Karmakar, T. & Taraphder, S. Understanding proton affinity of tyrosine sidechain in hydrophobic confinement#. *Journal of Chemical Sciences* **124**, 59-63, doi:10.1007/s12039-011-0196-y (2012).

7 Best, R. B. *et al.* Optimization of the Additive CHARMM All-Atom Protein Force Field Targeting Improved Sampling of the Backbone ϕ, ψ and Side-Chain $\chi 1$ and $\chi 2$ Dihedral Angles. *Journal of Chemical Theory and Computation* **8**, 3257-3273, doi:10.1021/ct300400x (2012).
