# Supplementary material for: Role of hydrogen bond alternation and charge transfer states in photoactivation of the Orange Carotenoid Protein
Source: Commun Biol. 2021 May 10;4:539. doi: 10.1038/s42003-021-02022-3 (PMC8110590; doi:10.1038/s42003-021-02022-3)
Supplement: Supplementary file 2 — Description of Additional Supplementary Files [file 42003_2021_2022_MOESM2_ESM.pdf]

Description of Additional Supplementary Files

File name: Supplementary Data 1

Description: Figure_1.xlsx. Normalized absorption spectra of different OCP variants in 0.8 M phosphate, presented in Figure 1. The tabs are labeled according to the panels in the figure.

File name: Supplementary Data 2

Description: Figure_2.xlsx. Time-courses of the dihedral angle (χ2) between the peptide backbone and the indole ring of Trp-201 as revealed by molecular dynamics simulations of OCP^{WW} with different initial conformation of Trp-201.

File name: Supplementary Data 3

Description: Figure_3.rar. Archive contains transient absorption spectra of WT OCP and OCP^{WW} at different time delays.

File name: Supplementary Data 4

Description: Figure_5.xlsx. Calculated and measured Raman spectra of ECN in regular and protonated form. Normalized steady-state absorption of ECN and CAN oxocarbenium ions obtained by protonation of carotenoids using 1 M trifluoroacetic acid (TFAA) in chloroform. The tabs are labeled according to the panels in the figure.
